# Supplementary material for: Empirical Assessment of Sequence-Based Predictions of Intrinsically Disordered Regions Involved in Phase Separation
Source: Biomolecules. 2025 Jul 25;15(8):1079. doi: 10.3390/biom15081079 (PMC12383833; doi:10.3390/biom15081079)
Supplement: Supplementary file 1 [file biomolecules-15-01079-s001.zip › biomolecules-3746426-supplementary.pdf]

Supplement

Xuantai Wu[1], Kui Wang[2], Gang Hu[2], and Lukasz Kurgan[3*]

[1]School of Mathematical Sciences and LPMC, Nankai University, Tianjin 300071, China

[2]NITFID, School of Statistics and Data Science, LPMC and KLMDASR, Nankai University, Tianjin, China

[3]Department of Computer Science, Virginia Commonwealth University, Richmond, VA 23284, USA

*corresponding author: lkurgan@vcu.edu; +1 804-827-3986

Test Dataset

Each test protein is represented by 3 consecutive lines:

1. >UniProt Accession Number;DisProt ID (if available)
2. AA sequence
3. Residue type annotations: P (residues in the experimentally annotated disordered regions associated with the Phase separation function); D (residues in the experimentally annotated Disordered regions that do not have the phase separation associated functions and have 1 or no non-phase separation associated function annotations – they are excluded from the analysis of predictive performance because their potential involvement in phase separation or lack thereof could be considered unclear); F (residues in the experimentally annotated disordered regions with 2 or more non-phase separation associated Functions); S (residues in the experimentally annotated Structured proteins); and X (Residues without disorder/structure/phase separation annotations, which are excluded from the analysis of predictive performance).

```
>P21707; DP04144
MVSASHPEALAAPVTTVATLVPHNATEPASPGEGKEDAFSKLKQKFMNELHKIPLPPWALIAIAIVAVLLVVTCCFCVCKKCLFKKKNKKKGKEKGGKNAINMKDVKDLGKTMKDQALKDDDAETGLTDGEEKEEPKEEE
KLGKLQYSLDYDFQNNQLLVGIIQAAELPALDMGGTSDPYVKVFLLPDKKKKFETKVHRKTLNPVFNEQFTFKVPYSELGGKTLVMAVYDFDRFSKHDIIGEFKVPMNTVDFGHVTEEWRDLQSAEKEEQEKLGDICFSL
RYVPTAGKLTVVILEAKNLKKMDVGGLSDPYVKIHLMQNGKRLKKKKTTIKKNTLNPYYNESFSFEVPFEQIQKVQVVVTVLDYDKIGKNDAIGKVFVGYNSTGAELRHWSDMLANPRRPIAQWHTLQVEEEVDAMLAVK
K
XXXXXXXXXXXXXXXXXXXXXXXXXXXXXXXXXXXXXXXXXXXXXXXXXXXXXXXXXXXXXXXXXXXXXXXXXXXXXXXPPPPPPPPPPPPPPPPPPPPPPPPPPPPPPPPPPPPPPPPPPPPPPPPPPPPPPPPPPPPPP
PPXXXXXXXXXXXXXXXXXXXXXXXXXXXXXXXXXXXXXXXXXXXXXXXXXXXXXXXXXXXXXXXXXXXXXXXXXXXXXXXXXXXXXXXXXXXXXXXXXXXXXXXXXXXXXXXXXXXXXXXXXXXXXXXXXXXXXXXXXXXX
XXXXXXXXXXXXXXXXXXXXXXXXXXXXXXXXXXXXXXXXXXXXXXXXXXXXXXXXXXXXXXXXXXXXXXXXXXXXXXXXXXXXXXXXXXXXXXXXXXXXXXXXXXXXXXXXXXXXXXXXXXXXXXXXXXXXXXXXXXXXX
X
>Q14444; DP02916
MPSATSHSGSGSKSSGPPPPSGSSGSEAAAGAGAAAPASQHPATGTGAVQTEAMKQILGVIDKKLRNLEKKKGKLDDYQERMNKGERLNQDQLDAVSKYQEVTNNLEFAKELQRSFMALSQDIQKTIKKTARREQLMREE
AEQKRLKTVLELQYVLDKLGDDEVRTDLKQGLNGVPILSEEELSLLDEFYKLVDPERDMSLRLNEQYEHASIHLWDLLEGKEKPVCGTTYKVLKEIVERVFQSNYFDSTHNHQNGLCEEEEAASAPAVEDQVPEAEPEPA
EEYTEQSEVESTEYVNRQFMAETQFTSGEKEQVDEWTVETVEVVNSLQQQPQAASPSVPEPHSLTPVAQADPLVRRQRVQDLMAQMQGPYNFIQDSMLDFENQTLDPAIVSAQPMNPTQNMDMPQLVCPPVHSESRLAQP
NQVPVQPEATQVPLVSSTSEGYTASQPLYQPSHATEQRPQKEPIDQIQATISLNTDQTTASSSLPAASQPQVFQAGTSKPLHSSGINVNAAPFQSMQTVFNMNAPVPPVNEPETLKQQNQYQASYNQSFSSQPHQVEQTE
LQQEQLQTVVGTYHGSPDQSHQVTGNHQQPPQQNTGFPRSNQPYYNSRGVSRGGSRGARGLMNGYRGPANGFRGGYDGYRPSFSNTPNSGYTQSQFSAPRDYSGYQRDGYQQNFKRGSGQSGPRGAPRGRGGPPRPNRGM
PQMNTQQVN
XXXXXXXXXXXXXXXXXXXXXXXXXXXXXXXXXXXXXXXXXXXXXXXXXXXXXXXXXXXXXXXXXXXXXXXXXXXXXXXXXXXXXXXXXXXXXXXXXXXXXXXXXXXXXXXXXXXXXXXXXXXXXXXXXXXXXXXXXXXX
XXXXXXXXXXXXXXXXXXXXXXXXXXXXXXXXXXXXXXXXXXXXXXXXXXXXXXXXXXXXXXXXXXXXXXXXXXXXXXXXXXXXXXXXXXXXXXXXXXXXXXXXXXXXXXXXXXXXXXXXXXXXXXXXXXXXXXXXXXXX
XXXXXXXXXXXXXXXXXXXXXXXXXXXXXXXXXXXXXXXXXXXXXXXXXXXXXXXXXXXXXXXXXXXXXXXXXXXXXXXXXXXXXXXXXXXXXXXXXXXXXXXXXXXXXXXXXXXXXXXXXXXXXXXXXXXXXXXXXXXX
XXXXXXXXXXXXXXXXXXXXXXXXXXXXXXXXXXXXXXXXXXXXXXXXXXXXXXXXXXXXXXXXXXXXXXXXXXXXXXXXXXXXXXXXXXXXXXXXXXXXXXXXXXXXXXXXXXXXXXXXXXXXXXXXXXXXXXXXXXXX
XXXXXXXXXXXXXXXXXXXXXXXXXXXXXXXXXXXXXXXXXXXXXXPPPPPPPPPPPPPPPPPPPPPPPPPPPPPPPPPPPPPPPPPPPPPPPPPPPPPPPPPPPPPPPPPPPPPPPPPPPPPPPPPPPPPPPPPPPPPP
PPPPPPPPP
>Q61474; DP04172
METDAPQPGLASPDSPHDPCKMFIGGLSWQTTQEGLREYFGQFGEVKECLVMRDPLTKRSRGFGFVTFMDQAGVDKVLAQSRHELDSKTIDPKVAFPRRAQPKMVTRTKKIFVGGLSVNTTVEDVKHYFEQFGKVDDAML
MFDKTTNRHRGFGFVTFESEDIVEKVCEIHFHEINNKMVECKKAQPKEVMSPTGSARGRSRVMPYGMDAFMLGIGMLGYPGFQATTYASRSYTGLAPGYTYQFPEFRVERSPLPSAPVLPELTAIPLTAYGPMAAAAAAA
AVVRGTGSHPWTMAPPPGSTPSRTGGFLGTTSPGPMAELYGAANQDSGVSSYISAASPAPSTGFGHSLGGPLIATAFTNGYH
XXXXXXXXXXXXXXXXXXXXXXXXXXXXXXXXXXXXXXXXXXXXXXXXXXXXXXXXXXXXXXXXXXXXXXXXXXXXXXXXXXXXXXXXXXXXXXXXXXXXXXXXXXXXXXXXXXXXXXXXXXXXXXXXXXXXXXXXXXXX
XXXXXXXXXXXXXXXXXXXXXXXXXXXXXXXXXXXXXXXXXXXXDDDDDDDDDPPPPPPPPPPPPPPPPPPPPPPPPPPPPPPPPPPPPPPPPPPPPPPPPPPPPPPPPPPPPPPPPPPPPPPPPPPPPPPPPPPPPPPPPPP
PPPPPPPPPPPPPPPPPPPPPPPPPPPPPPPPPPPPPPPPPPPPPPPPPPPPPPPPPPPPPPPPPPPPPPPPPPPPPPPPPP
```

>Q9BP37; DP01094
MTYMCSILICLVLILCARGAEADDNGNYGNGMASVRTQGNTYDDLASLISYLTRHSFRRPFHECALCYSITDPGERQRCIDMYCSYTN
XXXXXXXXXXXXXXXXXXXXXFFFFFFFFPPPPPPPPPPPPPPPPPPPPPPPPPPPPPPPPPPPPDDDDDDDDDDDDDDDDDDDDDDD
>P08621; DP02171
MTQFLPPNLLALFAPRDPIPYLPPLEKLPHEKHHNQPYCGIAPYIREFEDPRDAPPPTRAETREERMERKRREKIERRQQEVETELKMWDPHNDPNAQGDAFKTLFVARVNYDTTESKLRREFEVYGPIKRIHMVYSKRS
GKPRGYAFIEYEHERDMHSAYKHADGKKIDGRRVLVDVERGRTVKGWRPRRLGGGLGGTRRGGADVNIRHSGRDDTSRYDERPGPSPLPHRDRDRDRERERRERSRERDKERERRRSRSRDRRRRSRSRDKEERRRSRER
SKDKDRDRKRRSSRSRERARRERERKEELRGGGGDMAEPSEAGDAPPDDGPPGELGPDGPDGPEEKGRDRDRERRRSHRSERERRRDRDRDRDRDREHKRGERGSERGRDEARGGGGGQDNGLEGLGNDSRDMYMESEGG
DGYLAPENGYLMEAAPE
DDDDDDDDDDDDDDDDDDDDDDDDDDDDDDDDDDDDDDDDDDDDDDDDDDDDDDDDDDDDDDDDDDDDDDDDDDDDDDDDDDDDDDDDDDDDDDDDDDDDXXXXXXXXXXXXXXXXXXXXXXXXXXXXXXXXXXXXXXXX
XXXXXXXXXXXXXXXXXXXXXXXXXXXXXXXXXXXXXXXXXXXXXDDDDDDDDDDDDDDDDDDDDDDDDDDDDDDDDDDDDDDDDDDDDDDDDDDDDDDDDDDDDDDDDDDDPPPPPPPPPPPPPPPPPPPPPPPPPPPPPPPPPPPPP
PPPPPPPPPPPPPPPPPPPPPPPPPPPPPPPPPDDDDDDDDPPPPPPPPPPPPPPPPPPPPPPPPPPPPPPPPPPPPPPPPPPPPPPPPPPPPPPPPPPPPPPPPPPPPPPPPPPPPPPPPPPPPPPPDDDDDDDDDDDDD
DDDDDDDDDDDDDDDDD
>P0DTC9; DP03212
MSDNGPQNQRNAPRITFGGPSDSTGSNQNGERSGARSKQRRPQGLPNNTASWFTALTQHGKEDLKFPRGQGVPINTNSSPDDQIGYYRRATRRIRGGDGKMKDLSPRWYFYYLGTGPEAGLPYGANKDGIIWVATEGALN
TPKDHIGTRNPANNAAIVLQLPQGTTLPKGFYAEGSRGGSQASSRSSSRSRNSSRNSTPGSSRGTSPARMAGNGGDAALALLLLDRLNQLESKMSGKGQQQQGQTVTKKSAAEASKKPRQKRTATKAYNVTQAFGRRGPE
QTQGNFGDQELIRQGTDYKHWPQIAQFAPSASAFFGMSRIGMEVTPSGTWLTYTGAIKLDDKDPNFKDQVILLNKHIDAYKTFPPTEPKKDKKKKADETQALPQRQKKQQTVTLLPAADLDDFSKQLQQSMSSADSTQA
PPPPPPPPPPPPPPPPPPPPPPPPPPPPPPPPPPPPPPPPPPPPPPPPPPPPPPPPPPPPPPPPPPPPPPPPPPPPPPPPPPPPPPPPPPPPPPPPPPPPPPPPPPPPPPPPPPPPPPPPPPPPPPPPPPPPPPPPP
PPPPPPPPPPPPPPPPPPPPPPPPPPPPPPPPPPPPPPPPPPPPPPPPPPPPPPPPPPPPPPPPPPPPPPPPPPPPPPPPPPPPPPPPPPPPPPPPPPPPPPPPPPPPPPPPPPPPFDDDDDDDDDXXXXXXXXXXXXXX
XXXXXXXXXXXXXXXXXXXXXXXXXXXXXXXXXXXXXXXXXXXXXXXXXXXXXXXXXXXXXXXXXXXXXXXXXXXXXXXXDDDDDDDPPPPPPPPPPPPPPPPPPPPPPPPPPPPPPPPPPPPPPPPPPPPPPPPPPP
>P25054; DP00519
MAAASYDQLLKQVEALKMENSNLRQELEDNSNHLTKLETEASNMKEVLKQLQGSIEDEAMASSGQIDLLERLKELNLDSSNFPGVKLRSKMSLRSYGSREGSVSSRSGECSPVPMGSFPRRGFVNGSRESTGYLEELEKE
RSLLLADLDKEEKEKDWYYAQLQNLTKRIDSLPLTENFSLQTDMTRRQLEYEARQIRVAMEEQLGTCQDMEKRAQRRIARIQQIEKDILRIRQLLQSQATEAERSSQNKHETGSHDAERQNEGQGVGEINMATSGNGQGS
TTRMDHETASVLSSSSTHSAPRRLTSHLGTKVEMVYSLLSMLGTHDKDDMSRTLLAMSSSQDSCISMRQSGCLPLLIQLLHGNDKDSVLLGNSRGSKEARARASAALHNIIHSQPDDKRGRREIRVLHLLEQIRAYCETC
WEWQEAHEPGMDQDKNPMPAPVEHQICPAVCVLMKLSFDEEHRHAMNELGGLQAIAELLQVDCEMYGLTNDHYSITLRRYAGMALTNLTFGDVANKATLCSMKGCMRALVAQLKSESEDLQQVIASVLRNLSWRADVNSK
KTLREVGSVKALMECALEVKKESTLKSVLSALWNLSAHCTENKADICAVDGALAFLVGTLTYRSQTNTLAIIESGGGILRNVSSLIATNEDHRQILRENNCLQTLLQHLKSHSLTIVSNACGTLWNLSARNPKDQEALWD
MGAVSMLKNLIHSKHKMIAMGSAAALRNLMANRPAKYKDANIMSPGSSLPSLHVRKQKALEAELDAQHLSETFDNIDNLSPKASHRSKQRHKQSLYGDYVFDTNRHDDNRSDNFNTGNMTVLSPYLNTTVLPSSSSSRGS
LDSSRSEKDRSLERERGIGLGNYHPATENPGTSSKRGLQISTTAAQIAKVMEEVSAIHTSQEDRSSGSTTELHCVTDERNALRRSSAAHTHSNTYNFTKSENSNRTCSMPYAKLEYKRSSNDSLNSVSSSDGYGKRGQMK
PSIESYSEDDESKFCSYGQYPADLAHKIHSANHMDDNDGELDTPINYSLKYSDEQLNSGRQSPSQNERWARPKHIIEDEIKQSEQRQSRNQSTTYPVYTESTDDKHLKFQPHFGQQECVSPYRSRGANGSETNRVGSNHG
INQNVSQSLCQEDDYEDDKPTNYSERYSEEEQHEEEERPTNYSIKYNEEKRHVDQPIDYSLKYATDIPSSQKQSFSFSKSSSGQSSKTEHMSSSSENTSTPSSNAKRQNQLHPSSAQSRSGQPQKAATCKVSSINQETIQ
TYCVEDTPICFSRCSSLSSLSSAEDEIGCNQTTQEADSANTLQIAEIKEKIGTRSAEDPVSEVPAVSQHPRTKSSRLQGSSLSSESARHKAVEFSSGAKSPSKSGAQTPKSPPEHYVQETPLMFSRCTSVSSLDSFESRS
IASSVQSEPCSGMVSGIISPSDLPDSPGQTMPPSRSKTPPPPPQTAQTKREVPKNKAPTAEKRESGPKQAAVNAAVQRVQVLPDADTLLHFATESTPDGFSCSSSLSALSLDEPFIQKDVELRIMPPVQENDNGNETESE
QPKESNENQEKEAEKTIDSEKDLLDDSDDDDIEILEECIISAMPTKSSRKAKKPAQTASKLPPPVARKPSQLPVYKLLPSQNRLQPQKHVSFTPGDDMPRVYCVEGTPINFSTATSLSDLTIESPPNELAAGEGVRGGAQ
SGEFEKRDTIPTEGRSTDEAQGGKTSSVTIPELDDNKAEEGDILAECINSAMPKGKSHKPFRVKKIMDQVQQASASSSAPNKNQLDGKKKKPTSPVKPIPQNTEYRTRVRKNADSKNNLNAERVFSDNKDSKKQNLKNNS
KVFNDKLPNNEDRVRGSFAFDSPHHYTPIEGTPYCFSRNDSLSSLDFDDDDVDLSREKAELRKAKENKESEAKVTSHTELTSNQQSANKTQAIAKQPINRGQPKPILQKQSTFPQSSKDIPDRGAATDEKLQNFAIENTP
VCFSHNSSLSSLSDIDQENNNKENEPIKETEPPDSQGEPSKPQASGYAPKSFHVEDTPVCFSRNSSLSSLSIDSEDDLLQECISSAMPKKKKPSRLKGDNEKHSPRNMGGILGEDLTLDLKDIQRPDSEHGLSPDSENFD
WKAIQEGANSIVSSLHQAAAAACLSRQASSDSDSILSLKSGISLGSPFHLTPDQEEKPFTSNKGPRILKPGEKSTLETKKIESESKGIKGGKKVYKSLITGKVRSNSEISGQMKQPLQANMPSISRGRTMIHIPGVRNSS
SSTSPVSKKGPPLKTPASKSPSEGQTATTSPRGAKPSVKSELSPVARQTSQIGGSSKAPSRSGSRDSTPSRPAQQPLSRPIQSPGRNSISPGRNGISPPNKLSQLPRTSSPSTASTKSSGSGKMSYTSPGRQMSQQNLTK
QTGLSKNASSIPRSESASKGLNQMNNGNGANKKVELSRMSSTKSSGSESDRSERPVLVRQSTFIKEAPSPTLRRKLEESASFESLSPSSRPASPTRSQAQTPVLSPSLPDMSLSTHSSVQAGGWRKLPPNLSPTIEYNDG
RPAKRHDIARSHSESPSRLPINRSGTWKREHSKHSSSLPRVSTWRRTGSSSSILSASSESSEKAKSEDEKHVNSISGTKQSKENQVSAKGTWRKIKENEFSPTNSTSQTVSSGATNGAESKTLIYQMAPAVSKTEDVWVR
IEDCPINNPRSGRSPTGNTPPVIDSVSEKANPNIKDSKDNQAKQNVGNGSVPMRTVGLENRLNSFIQVDAPDQKGTEIKPGQNNPVPVSETNESSIVERTPFSSSSSSKHSSPSGTVAARVTPFNYNPSPRKSSADSTSA
RPSQIPTPVNNNTKKRDSKTDSTESSGTQSPKRHSGSYLVTSV
XXXXXXXXXXXXXXXXXXXXXXXXXXXXXXXXXXXXXXXXXXXXXXXXXXXXXXXXXXXXXXXXXXXXXXXXXXXXXXXXXXXXXXXXXXXXXXXXXXXXXXXXXXXXXXXXXXXXXXXXXXXXXXXXXXXXXXXXXX
XXXXXXXXXXXXXXXXXXXXXXXXXXXXXXXXXXXXXXXXXXXXXXXXXXXXXXXXXXXXXXXXXXXXXXXXXXXXXXXXXXXXXXXXXXXXXXXXXXXXXXXXXXXXXXXXXXXXXXXXXXXXXXXXXXXXXXXXXXXX
XXXXXXXXXXXXXXXXXXXXXXXXXXXXXXXXXXXXXXXXXXXXXXXXXXXXXXXXXXXXXXXXXXXXXXXXXXXXXXXXXXXXXXXXXXXXXXXXXXXXXXXXXXXXXXXXXXXXXXXXXXXXXXXXXXXXXXXXXXXX
XXXXXXXXXXXXXXXXXXXXXXXXXXXXXXXXXXXXXXXXXXXXXXXXXXXXXXXXXXXXXXXXXXXXXXXXXXXXXXXXXXXXXXXXXXXXXXXXXXXXXXXXXXXXXXXXXXXXXXXXXXXXXXXXXXXXXXXXXXXX
XXXXXXXXXXXXXXXXXXXXXXXXXXXXXXXXXXXXXXXXXXXXXXXXXXXXXXXXXXXXXXXXXXXXXXXXXXXXXXXXXXXXXXXXXXXXXXXXXXXXXXXXXXXXXXXXXXXXXXXXXXXXXXXXXXXXXXXXXXXX
XXXXXXXXXXXXXXXXXXXXXXXXXXXXXXXXXXXXXXXXXXXXXXXXXXXXXXXXXXXXXXXXXXXXXXXXXXXXXXXXXXXXXXXXXXXXXXXXXXXXXXXXXXXXXXXXXXXXXXXXXXXXXXXXXXXXXXXXXXXX
XXXXXXXXXXXXXXXXXXXXXXXXXXXXXXXXXXXXXXXXXXXXXXXXXXXXXXXXXXXXXXXXXXXXXXXXXXXXXXXXXXXXXXXXXXXXXXXXXXXXXXXXXXXXXXXXXXXXXXXXXXXXXXXXXXXXXXXXXXXX
XXXXXXXXXXXXXXXXXXXXXXXXXXXXXXXXXXXXXXXXXXXXXXXXXXXXXXXXXXXXXXXXXXXXXXXXXXXXXXXXXXXXXXXXXXXXXXXXXXXXXXXXXXXXXXXXXXXXXXXXXXXXXXXXXXXXXXXXXXXX
XXXXXXXXXXXXXXXXXXXXXXXXXXXXXXXXXXXXXXXXXXXXXXXXXXXXXXXXXXXXXXXXXXXXXXXXXXXXXXXXDDDDDDDDDDDDDDDDDDDDDDDDDDDDDDDDDDDDDDDDDDDDDDDDDDDDDDDDDDDD
DDDDDDDDDDDDDDDDDDDDDDDDDDDDDDDDDDDDDDDDDDDDDDDDDDDDDDDDDDDDDDDDDDDDDDDDDDDDDDDDDDDDDDDDDDDDDDDDDDDDDDDDDPPPPPPPPPPPPPPPPPPPPPPPPPPPPPPPPPPPPP
PPPPPPPPPPPPPPPPPPPPPPPPPPPPPPPPPPPPPPPPPPPPPPPPPPPPPPPPPPPPPPPPPPPPPPPPPPPPPPPPPPPPPPPPPPPPPPPPPPPPPPPPPPPPPPPPPPPPPPPPPPPPPPPPPPPPPPPPPPPP
PPPPPPPPPPPPDDDDDDDDDDDDDDDDDDDDDDDDDDDDDDDDDDDDDDDDDDDDDDDDDDDDDDDDDDDDDDDDDDDDDDDDDDDDDDDDDDDDDDDDDDDDDDDDDDDDDDDDDDDDDDDDDDDDDDDDDDDDDD
DDDDDDDDDDDDDDDDDDDDDDDDDDDDDDDDDDDDDDDDDDDDDDDDDDDDDDDDDDDDDDDDDXXXXXXXXXXXXXXXXXXXXXXXXXXXXXXXXXXXXXXXXXXXXXXXXXXXXXXXXXXXXXXXXXXXXXXXXXXX

XXXXXXXXXXXXXXXXXXXXXXXXXXXXXXXXXXXXXXXXXXXXXXXXXXXXXXXXXXXXXXXXXXXXXXXXXXXXXXXXXXXXXXXXXXXXXXXXXXXXXXXXXXXXXXXXXXXXXXXXXXXXXXXXXXXXXXXXXX
XXXXXXXXXXXXXXXXXXXXXXXXXXXXXXXXXXXXXXXXXXXXXXXXXXXXXXXXXXXXXXXXXXXXXXXXXXXXXXXXXXXXXXXXXXXXXXXXXXXXXXXXXXXXXXXXXXXXXXXXXXXXXXXXXXXXXXXXXX
XXXXXXXXXXXXXXXXXXXXXXXXXXXXXXXXXXXXXXXXXXXXXXXXXXXXXXXXXXXXXXXXXXXXXXXXXXXXXXXXXXXXXXXXXXXXXXXXXXXXXXXXXXXXXXXXXXXXXXXXXXXXXXXXXXXXXXXXXX
XXXXXXXXXXXXXXXXXXXXXXXXXXXXXXXXXXXXXXXXXXXXXXXXXXXXXXXXXXXXXXXXXXXXXXXXXXXXXXXXXXXXXXXXXXXXXXXXXXXXXXXXXXXXXXXXXXXXXXXXXXXXXXXXXXXXXXXXXX
XXXXXXXXXXXXXXXXXXXXXXXXXXXXXXXXXXXXXXXXXXXXXXXXXXXXXXXXXXXXXXXXXXXXXXXXXXXXXXXXXXXXXXXXXXXXXXXXXXXXXXXXXXXXXXXXXXXXXXXXXXXXXXXXXXXXXXXXXX
XXXXXXXXXXXXXXXXXXXXXXXXXXXXXXXXXXXXXXXXXXXXXXXXXXXXXXXXXXXXXXXXXXXXXXXXXXXXXXXXXXXXXXXXXXXXXXXXXXXXXXXXXXXXXXXXXXXXXXXXXXXXXXXXXXXXXXXXXX
XXXXXXXXXXXXXXXXXXXXXXXXXXXXXXXXXXXXXXXXXXXXXXXXXXXXXXXXXXXXXXXXXXXXXXXXXXXXXXXXXXXXXXXXXXXXXXXXXXXXXXXXXXXXXXXXXXXXDDDDDDDDDDDDDDDDDDDD
DDDDDDDDDDDDDDDDDDDXXXXXXXXXXXXXXXXXXXXXXXX
>Q02199; DP02122
MFGLNKASSTPAGGLFGQASGASTGNANTGFSFGGTQTGQNTGPSTGGLFGAKPAGSTGGLGASFGQQQQQSQTNAFGGSATTGGGLFGNKPNNTANTGGGLFGANSNSNSGSLFGSNNAQTSRGLFGNNNTNNINNSSS
GMNNASAGLFGSKPAGGTSLFGNTSTSSAPAQNQGMFGAKPAGTSLFGNNAGNTTTGGGLFGSKPTGATSLFGSSNNNNNNNNSNNIMSASGGLFGNQQQQLQQQPQMQCALQNLSQLPITPMTRISELPPQIRQEIEQL
DQYIQKQVQISHHLKADTIDHDELIDSIPRDVAYLLKSESATSQYLKQDLKKISSFKSLIDEDLLDTQTFSVLLQQLLTPGSKISSNDLDKFFQKKIHLYEKKLEDYCRILSDIETAVNGIDTDLFGAPNNPNSTAITAD
LGSSEAENLLQLKTGLAAIVSTVIEEFTLFMDIAERIAVLHQKTKTLASLSI
PPPPPPPPPPPPPPPPPPPPPPPPPPPPPPPPPPPPPPPPPPPPPPPPPPPPPPPPPPPPPPPPPPPPPPPPPPPPPPPPPPPPPPPPPPPPPPPPPPPPPPPPPPPPPPPPPPPPPPPPPPPPPPPPPPPPPPPPPP
PPPPPPPPPPPPPPPPPPPPPPPPPPPPPPPPPPPPPPPPPPPPPPPPPPPPPPPPPPPPPPPPPPPPPPPPPPPPPPPPPPPPPPPPPPPPPPPPPPPPPPPPPPPPPDDDDDDDDDDDDDDDDDDDDXXXXXXXXXXX
XXXXXXXXXXXXXXXXXXXXXXXXXXXXXXXXXXXXXXXXXXXXXXXXXXXXXXXXXXXXXXXXXXXXXXXXXXXXXXXXXXXXXXXXXXXXXXXXXXXXXXXXXXXXXXXXXXXXXXXXXXXXXXXDDDDDDDDDDDDD
DDDDDDDDXXXXXXXXXXXXXXXXXXXXXXXXXXXXXXXXXXXXXXXXXXXX
>Q09472; DP00633
MAENVVEPGPPSAKRPKLSSPALSASASDGTDFGSLFDLEHDLPDELINSTELGLTNGGDINQLQTSLGMVQDAASKHKQLSELLRSGSSPNLNMGVGGPGQVMASQAQQSSPGLGLINSMVKSPMTQAGLTSPNMGMGT
SGPNQGPTQSTGMMNSPVNQPAMGMNTGMNAGMNPGMLAAGNGQGIMPNQVMNGSIGAGRGRQNMQYPNPGMGSAGNLLTEPLQQGSPQMGGQTGLRGPQPLKMGMMNNPNPYGSPYTQNPGQQIGASGLGLQIQTKTVL
SNNLSPFAMDKKAVPGGGMPNMGQQPAPQVQQPGLVTPVAQGMGSGAHTADPEKRKLIQQQLVLLLHAHKCQRREQANGEVRQCNLPHCRTMKNVLNHMTHCQSGKSCQVAHCASSRQIISHWKNCTRHDCPVCLPLKNA
GDKRNQQPILTGAPVGLGNPSSLGVGQQSAPNLSTVSQIDPSSIERAYAALGLPYQVNQMPTQPQVQAKNQQNQQPGQSPQGMRPMSNMSASPMGVNGGVGVQTPSLLSDSMLHSAINSQNPMMSENASVPSLGPMPTAA
QPSTTGIRKQWHEDITQDLRNHLVHKLVQAIFPTPDPAALKDRRMENLVAYARKVEGDMYESANNRAEYYHLLAEKIYKIQKELEEKRRTRLQKQNMLPNAAGMVPVSMNPGPNMGQPQPGMTSNGPLPDPSMIRGSVPN
QMMPRITPQSGLNQFGQMSMAQPPIVPRQTPPLQHHGQLAQPGALNPPMGYGPRMQQPSNQGQFLPQTQFPSQGMNVTNIPLAPSSGQAPVSQAQMSSSSCPVNSPIMPPGSQGSHIHCPQLPQPALHQNSPSPVPSRTP
TPHHTPPSIGAQQPPATTIPAPVPTPPAMPPGPQSQALHPPPRQTPTPPTTQLPQQVQPSLPAAPSADQPQQQPRSQQSTAASVPTPTAPLLPPQPATPLSQPAVSIEGQVSNPPSTSSTEVNSQAIAEKQPSQEVKMEA
KMEVDQPEPADTQPEDISESKVEDCKMESTETEERSTELKTEIKEEEDQPSTSATQSSPAPGQSKKKIFKPEELRQALMPTLEALYRQDPESLPFRQPVDPQLLGIPDYFDIVKSPMDLSTIKRKLDTGQYQEPWQYVDD
IWLMFNNAWLYNRKTSRVYKYCSKLSEVFEQEIDPVMQSLGYCCGRKLEFSPQTLCCYGKQLCTIPRDATYYSYQNRYHFCEKCFNEIQGESVSLGDDPSQPQTTINKEQFSKRKNDTLDPELFVECTECGRKMHQICVL
HHEIIWPAGFVCDGCLKKSARTRKENKFSAKRLPSTRLGTFLENRVNDFLRRQNHPESGEVTVRVVHASDKTVEVKPGMKARFVDSGEMAESFPYRTKALFAFEEIDGVDLCFFGMHVQEYGSDCPPPNQRRVYISYLDS
VHFFRPKCLRTAVYHEILIGYLEYVKKLGYTTGHIWACPPSEGDDYIFHCHPPDQKIPKPKRLQEWYKKMLDKAVSERIVHDYKDIFKQATEDRLTSAKELPYFEGDFWPNVLEESIKELEQEEEERKREENTSNESTDV
TKGDSKNAKKKNNKKTSKNKSSLSRGNKKKPGMPNVSNDLSQKLYATMEKHKEVFFVIRLIAGPAANSLPPIVDPDPLIPCDLMDGRDAFLTLARDKHLEFSSLRRAQWSTMCMLVELHTQSQDRFVYTCNECKHHVETR
WHCTVCEDYDLCITCYNTKNHDHKMEKLGLGLDDESNNQQAAATQSPGDSRRLSIQRCIQSLVHACQCRNANCSLPSCQKMKRVVQHTKGCKRKTNGGCPICKQLIALCCYHAKHCQENKCPVPFCLNIKQKLRQQQLQH
RLQQAQMLRRRMASMQRTGVVGQQQGLPSPTPATPTTPTGQQPTTPQTPQPTSQPQPTPPNSMPPYLPRTQAAGPVSQGKAAGQVTPPTPPQTAQPPLPGPPPAAVEMAMQIQRAAETQRQMAHVQIFQRPIQHQMPPMT
PMAPMGMNPPPMTRGPSGHLEPGMGPTGMQQQPPWSQGGLPQPQQLQSGMPRPAMMSVAQHGQPLNMAPQPGLGQVGISPLKPGTVSQQALQNLLRTLRSPSSPLQQQQVLSILHANPQLLAAFIKQRAAKYANSNPQPI
PGQPGMPQGQPGLQPPTMPGQQGVHSNPAMQNMNPMQAGVQRAGLPQQQPQQQLQPPMGGMSPQAQQMNMNHNTMPSQFRDILRRQQMMQQQQQQGAGPGIGPGMANHNQFQQPQGVGYPPQQQQRMQHHMQQMQQGNMG
QIGQLPQALGAEAGASLQAYQQRLLQQQMGSPVQPNPMSPQQHMLPNQAQSPHLQGQQIPNSLSNQVRSPQPVPSPRPQSQPPHSSPSPRMQPQPSPHHVSPQTSSPHPGLVAAQANPMEQGHFASPDQNSMLSQLASNP
GMANLHGASATDLGLSTDNSDLNSNLSQSTLDIH
DDDDDDDDDDDDDDDDDDDDDDDDDDDDDDDDDDDDDDDDDDDDDDDDDDDDDDDDDDDDDDDDDDDDDDDDDDDDDDDDDDDDDDDDDDDDDDDDDDDDDDDDDDDDDDDDDDDDDDDDDDDDDDDDDDDDDDDDDD
DDDDDDDDDDDDDDDDDDDDDDDDDDDDDDDDDDDDDDDDDDDDDDDDDDDDDDDDDDDDDDDDDDDDDDDDDDDDDDDDDDDDDDDDDDDDDDDDDDDDDDDDDDDDDDDPPPPPPPPPPPPPPPPPPPPPPPPPPPPPP
PPPPPPPPPPPPPPPPPPPPPPPPPPPPPPPPPPPPPPPPPPPPPPPPPPPPPPPPPPPPPPPPPPPPPPPPPPPPPPPPPPPPPPPPPPPPPPPPPPPPPPPPPPPPPPPPPPPPPPPPPPPPPPPPPPPPPPPPPP
PPPPPPPPPPPPPPPPPPPPPPPPPPPPPPPPPPPPPPPPPPPPPPPPPPPPPPPPPPPPPPPPPPPPPPPPPPPPPPPPPPPPPPPPPPPPPPPPPPPPPPPPPPPPPPPPPPPPPPPPPPPPPPPPPPPPPDDDDDDD
DDDDDDDDDDDDDDDDDDDDDDDDDDDDDDDDDDDDDDDDDDDDDDDDDDDDDDDDDDDDDDDDDDDDDDDDDDDDDDDDDDDDDDDDDDDDDDDDDDDDDDDDDDDDDDDDDDDDDDDDDDDDDDDDDDDDDDDDDD
DDDDDDDDDDDDDDDDDDDDDDDDDDDDDDDDDDDDDDDDDDDDDDDDDDDDDDDDDDDDDDDDDDDDDDDDDDDDDDDDDDDDDDDDDDDDDDDDDDDDDDDDDDDDDDDDDDDDDDDDDDDDDDDDDDDDDDDDDD
DDDDDDDDDDDDDDDDDDDDDDDDDDDDDDDDDDDDDDDDDDDDDDDDDDDDDDDDDDDDDDDDDDDDDDDDDDDDDDDDDDDDDDDDDDDDDDDDDDDDDDDDDDDDDDDDDDDDDDDDDDDDDDDDDDDDDDDDDD
DDDDDDDDDDDDDDDDDDDDDDDDDDDDDDDDDDDDDDDDDDDDDDDDDDDDDDDDDDDDDDDDDXXXXXXXXXXXXXXXXXXXXXXXXXXXXXXXXXXXXXXXXXXXXXXXXXXXXXXXXXXXXXXXXXXXXXXXXXXX
XXXXXXXXXXXXXXXXXXXXXXXXXXXXXXXXXXXXXXXXXXXXXXXXXXXXXXXXXXXXXXXXXXXXXXXXXXXXXXXXXXXXXXXXXXXXXXXXXXXXXXXXXXXXXXXXXXXXXXXXXXXXXXXXXXXXXXXXXX
XXXXXXXXXXXXXXXXXXXXXXXXXXXXXXXXXXXXXXXXXXXXXXXXXXXXXXXXXXXXXXXXXXXXXXXXXXXXXXXXXXXXXXXXXXXXXXXXXXXXXXXXXXXXXXXXXXXXXXXXXXXXXXXXXXXXXXXXXX
XXXXXXXXXXXXXXXXXXXXXXXXXXXXXXXXXXXXXXXXXXXXXXXXXXXXXXXXXXXXXXXXXXXXXXXXXXXXXXXXXXXXXXXXXXXXXXXXXXXXXXXXXXXXXXXDDDDDDDDDDDDDDDDDDDDD
DDDDDDDDDDDDDDDDDDDDDDDDDDDDDDDDDDDDDDDDDXXXXXXXXXXXXXXXXXXXXXXXXXXXXXXXXXXXXXXXXXXXXXXXXXXXXXXXXXXXXXXXXXXXXXXXXXXXXXXXXXXXXXXXXXXXDDDDDDDDDDDDDDDD
DDDDDDDDDDDDDDDDDDDDDDDDDDDDDDDDDDDDDDDDDDDDDDDDDDDDDDDDDDDDDDDDDDDDDDDDDDDDDDDDDDDDDDDDDDDDDDDDDDDDDDDDDDDDDDDDDDDDDDDDDDDDDDDDDDDDDDDDDD
DDDDDDDDDDDDDDDDDDDDDDDDDDDDDDDDDDDDDDDDDDDDDDDDDDDDDDDDDDDDDDDDDDDDDDDDDDDDDDDDDDDDDDDDDDDDDDDDDDDDDDDDDDDDDDDDDDDDDDDDDDDDDDDDDDDDDDDDD
DDDDDDDDDDDDDDDDDDDDDDDDDDDDDDDDDDDDDDDDDDDDDDDDDDDDDDDDDDDDDDDDDDDDDDDDDDDDDDDDDDDDDDDDDDDDDDDDDDDDDDDDDDDDDDDDDDDDDDDDDDDDDDDDDDDDDDDDDD
DDDDDDDDDDDDDDDDDDDDDDDDDDDDDDDDDDDDDDDDDDDDDDDDDDDDDDDDDDDDDDDDDDDDDDDDDDDDDDDDDDDDDDDDDDDDDDDDDDDDDDDDDDDDDDDDDDDDDDDDDDDDDDDDDDDDDDDDDD
DDDDDDDDDDDDDDDDDDDDDDDDDDDDDDDDDDDDDDDDDDDDDDDDDDDDDDDDDDDDDDDDDDDDDDDDDDDDDDDDDDDDDDDDDDDDDDDDDDDDDDDDDDDDDDDDDDDDDDDDDDDDDDDDDDDDDDDDDD
DDDDDDDDDDDDDDDDDDDDDDDDDDDDDDDDDDDDDDDDDDDDDDDDDDDDDDDDDDDDDDDDDDDDDDDDDDDDDDDDDDDDDDDDDDDDDDDDDDDDDDDDDDDDDDDDDDDDDDDDDDDDDDDDDDDDDDDDDD
DDDDDDDDDDDDDDDDDDDDDDDDDDDDDDDDDD

>Q13177; DP03447
MSDNGELEDKPPAPPVRMSSTIFSTGGKDPLSANHSLKPLPSVPEEKKPRHKIISIFSGTEKGSKKKEKERPEISPPSDFEHTIHVGFDAVTGEFTGMPEQWARLLQTSNITKLEQKKNPQAVLDVLKFYDSNTVKQKYL
SFTPPEKDGFPSGTPALNAKGTEAPAVVTEEEDDDEETAPPVIAPRPDHTKSIYTRSVIDPVPAPVGDSHVDGAAKSLDKQKKKTKMTDEEIMEKLRTIVSIGDPKKKYTRYEKIGQGASGTVFTATDVALGQEVAIKQI
NLQKQPKKELIINEILVMKELKNPNIVNFLDSYLVGDELFVVMEYLAGGSLTDVVTETCMDEAQIAAVCRECLQALEFLHANQVIHRDIKSDNVLLGMEGSVKLTDFGFCAQITPEQSKRSTMVGTPYWMAPEVVTRKAY
GPKVDIWSLGIMAIEMVEGEPPYLNENPLRALYLIATNGTPELQNPEKLSPIFRDFLNRCLEMDVEKRGSAKELLQHPFLKLAKPLSSLTPLIMAAKEAMKSNR
DDDDDDDDDDDDDDDDDDDDDDDDDDDDDDDDDDDDDDDDDDDDDDDDDDDDDDDDDDDDDDDDDDDDDDDDDDDDDDDDDDDDDDDDDDDDDDDDDDDDDDDDDDDDDDDDDDDDDDDDDDDDDDDDDDDDDDDDDDFF
FFFDDDDDDDDDDDDDDDDDDDDDDDDDDDDDDDDPPPPPPPPPPPPPPPPPPPPPPPDDDDDDDDDDDFFFFFFDDDDDDDDDDDDDDDDDDDDDDDXXXXXXXXXXXXXXXXXXXXXXXXXXXXXXXXXXXXXXXXXX
XXXXXXXXXXXXXXXXXXXXXXXXXXXXXXXXXXXXXXXXXXXXXXXXXXXXXXXXXXXXXXXXXXXXXXXXXXXXXXXXXXXXXXXXXXXXXXXXXXXXXXXXXXXXXDDDDDDDDDFFFFFXXXXXXXXXXXXXXXXX
XXXXXXXXXXXXXXXXXXXXXXXXXXXXXXXXXXXXXXXXXXXXXXXXXXXXXXXXXXXXXXXXXXXXXXXXXXXXXXXXXXXXXXXXXXXXXXXXXXXXXXXX
>Q92800; DP04135
MEIPNPPTSKCITYWKRKVKSEYMRLRQLKRLQANMGAKALYVANFAKVQEKTQILNEEWKKLRVQPVQSMKPVSGHPFLKKCTIESIFPGFASQHMLMRSLNTVALVPIMYSWSPLQQNFMVEDETVLCNIPYMGDEVK
EEDETFIEELINNYDGKVHGEEEMIPGSVLISDAVFLELVDALNQYSDEEEEGHNDTSDGKQDDSKEDLPVTRKRKRHAIEGNKKSSKKQFPNDMIFSAIASMFPENGVPDDMKERYRELTEMSDPNALPPQCTPNIDGP
NAKSVQREQSLHSFHTLFCRRCFKYDCFLHPFHATPNVYKRKNKEIKIEPEPCGTDCFLLLEGAKEYAMLHNPRSKCSGRRRRRHHIVSASCSNASASAVAETKEGDSDRDTGNDWASSSSEANSRCQTPTKQKASPAPP
QLCVVEAPSEPVEWTGAEESLFRVFHGTYFNNFCSIARLLGTKTCKQVFQFAVKESLILKLPTDELMNPSQKKKRKHRLWAAHCRKIQLKKDNSSTQVYNYQPCDHPDRPCDSTCPCIMTQNFCEKFCQCNPDCQNRFPG
CRCKTQCNTKQCPCYLAVRECDPDLCLTCGASEHWDCKVVSCKNCSIQRGLKKHLLLAPSDVAGWGTFIKESVQKNEFISEYCGELISQDEADRRGKVYDKYMSSFLFNLNNDFVVDATRKGNKIRFANHSVNPNCYAKV
VMVNGDHRIGIFAKRAIQAGEELFFDYRYSQADALKYVGIERETDVL
DDDDDDDDDDDDDDDDDDDDDDDDDDDDDDXXXXXXXXXXXXXXXXXXXXXXXXXXXXXXXXXXXXXXXXXXXXXXXXXXXXXXXXXXXXXXXXXXXXXXXXXXXXXXXXXXXXXXXXXXXXXXXXXXXXDDDDDDDDDDDDDDDD
DDDDDDDDDDDDDDDDDDDDDDDDDDDDDDDDDDDDDDDDDDDDDDDDDDDDDDDDDDDDDDDDDDDDDDDDDDDDDDDDDDDDDDDDDDDDDDDDDDDDDDDDDDDDDDDDDDDDDDDDDDDDDDDDDDDDDDDXXXXXXXXX
XXXXXXXXXXXXXXXXXXXXXXXXXXXXXXXXXXXXXXXXXXPPPPPPPPPPPPPPPPPPPPPPPPPPPPPPPPPPPPPPPPPPPPPPPPPPPPPPPPPPPPPPPPPPPPPPPPPPPPPPPPPPPPPPPPPPPPPPPPPPPPPP
PPPPPPPPPPPXXXXXXXXXXXXXXXXXXXXXXXXXXXXXXXXXXXXXXXXXXXXXXDDDDDDDDDDDDDDDDDDDDDDDDDDDDDDDDDDDDDDDDXXXXXXXXXXXXXXXXXXXXXXXXXXXXXXXXXXXXXXXXXXX
XXXXXXXXXXXXXXXXXXXXXXXXXXXXXXXXXXXXXXXXXXXXXXXXXXXXXXXXXXXXXXXXXXXXXXXXXXXXXXXXXXXXXXXXXXXXXXXXXXXXXXXXXXXXXXXXXXXXXXXXXXXXXXXXXXXXXXXXXXXXXX
XXXXXXXXXXXXXXXXXXXXXXXXXXXDDDDDDDDDDDDDDDDDDDD
>A0A2U8QNT7; DP03392
MSENIGHININANLQNVDRRDAAAIQSVERKLLGYNPPGGLASEAQSAAALNEGIGQPMNRGISTDIPAPADIDVDRGTASKDFGHVRFDVDLNQVRPEEAAALQAAESKIEGLAPSITVGGIGSAAQSMAAFNEREQSE
TGPFHPGIKATEPLPGPTYYQGVELSPSALPTYAPDVSVFPPSLSTNTSNVGAVPPSITTYSPDAGANDWERVYRKTTKTTQRIAIPGGIEDIVDEGKLGEAPRTNIRSTIGNVRMD
PPPPPPPPPPPPPPPPPPPPPPPPPPPPPPPPPPPPPPPPPPPPPPPPPPPPPPPPPPPPPPPPPPPPPPPPPPPPPPPPPPPPPPPPPPPPPPPPPPPPPPPPPPPPPPPPPPPPPPPPPPPPPPPPPPPPPPPPPPPPPP
PPPPPPPPPPPPPPPPPPPPPPPPPPPPPPPPPPPPPPPPPPPPPPPPPPPPPPPPPPPPPPPPPPPPPPPPPPPPPPPPPPPPPPPPPPPPPPPPPPPPPPPPPPPPPPPPPPPPP
>P42763; DP00667
MAEEIKNVPEQEVPKVATEESSAEVTDRGLFDFLGKKKDETKPEETPIASEFEQKVHISEPEPEVKHESLLEKLHRSDSSSSSSSEEEGSDGEKRKKKKEKKKPTTEVEVKEEEKKGFMEKLKEKLPGHKKPEDGSAVAA
APVVVPPPVEEAHPVEKKGILEKIKEKLPGYHPKTTVEEEKKDKE
PPPPPPPPPPPPPPPPPPPPPPPPPPPPPPPPPPPPPPPPPPPPPPPPPPPPPPPPPPPPPPPPPPPPPPPPPPPPPPPPPPPPPPPPPPPPPPPPPPPPPPPPPPPPPPPPPPPPPPPPPPPPPPPPPPPPPPPPPPPPPP
PPPPPPPPPPPPPPPPPPPPPPPPPPPPPPPPPPPPPPPPPPPPP
>P80723; DP00930
MGGKLSKKKKGYNVNDEKAKEKDKKAEGAATEEEGTPKESEPQAAAEPAEAKEGKEKPDQDAEGKAEEKEGEKDAAAAKEEAPKAEPEKTEGAAEAKAEPPKAPEQEQAAPGPAAGGEAPKAAEAAAAPAESAAPAAGEE
PSKEEGEPKKTEAPAAPAAQETKSDGAPASDSKPGSSEAAPSSKETPAATEAPSSTPKAQGPAASAEEPKPVEAPAANSDQTVTVKE
PPPPPPPPPPPPPPPPPPPPPPPPPPPPPPPPPPPPPPPPPPPPPPPPPPPPPPPPPPPPPPPPPPPPPPPPPPPPPPPPPPPPPPPPPPPPPPPPPPPPPPPPPPPPPPPPPPPPPPPPPPPPPPPPPPPPPPPPPPPPPP
PPPPPPPPPPPPPPPPPPPPPPPPPPPPPPPPPPPPPPPPPPPPPPPPPPPPPPPPPPPPPPPPPPPPPPPPPPPPPPPPPPPPPPP
>Q9C7W1; DP04199
MQYYENREKDYYEVAQGQRNGYGQSQSHNHEGYGQSQSRGGYGQIHNREGYNQNREGYSQSQSRPVYGLSPTLNHRSHGGFLDGLFKGQNGQKGQSGLGTFLGQHKSQEAKKSQGHGKLLGQHDQKKTHETNSGLNGLGM
FINNGEKKHRRKSEHKKKNKDGHGSGNESGSSSGSDSD
PPPPPPPPPPPPPPPPPPPPPPPPPPPPPPPPPPPPPPPPPPPPPPPPPPPPPPPPPPPPPPPPPPPPPPPPPPPPPPPPPPPPPPPPPPPPPPPPPPPPPPPPPPPPPPPPPPPPPPPPPPPPPPPPPPPPPPPPPPPPPP
PPPPPPPPPPPPPPPPPPPPPPPPPPPPPPPPPPPPPP
>Q9NP98; DP03529
MPLSGTPAPNKKRKSSKLIMELTGGGQESSGLNLGKKISVPRDVMLEELSLLTNRGSKMFKLRQMRVEKFIYENHPDVFSDSSMDHFQKFLPTVGGQLGTAGQGFSYSKSNGRGGSQAGGSGSAGQYGSDQQHHLGSGSG
AGGTGGPAGQAGRGGAAGTAGVGETGSGDQAGGEGKHITVFKTYISPWERAMGVDPQQKMELGIDLLAYGAKAELPKYKSFNRTAMPYGGYEKASKRMTFQMPKFDLGPLLSEPLVLYNQNLSNRPSFNRTPIPWLSSGE
PVDYNVDIGIPLDGETEEL
PPPPPPPPPPPPPPPPPPPPPPPPPPPPPPPPPPPPPPPPPPPPPPPPPPPPPPPPPPPPPPPPPPPPPPPPPPPPPPPPPPPPPPPPPPPPPPPPPPPPPPPPPPPPPPPPPPPPPPPPPPPPPPPPPPPPPPPPPPPPPP
PPPPPPPPPPPPPPPPPPPPPPPPPPPPPPPPPPPPPPPPPPPPPPPPPPPPPPPPPPPPPPPPPPPPPPPPPPPPPPPPPPPPPPPPPPPPPPPPPPPPPPPPPPPPPPPPPPPPPPPPPPPPPPPPPPPPPPPPPPPPPP
PPPPPPPPPPPPPPPPPPP
>Q9RFD6; DP02993
MSPKDLTIPTGADGEGSVQVHLDEADKITGAKVFAVYGKGGIGKSTTSSNLSAAFSILGKRVLQIGCDPKHDSTFTLTGSLVPTVIDVLKDVDFHPEELRPEDFVFEGFNGVMCVEAGGPPAGTGCGGYVVGQTVKLLKQ
HHLLDDTDVVIFDVLGDVVCGGFAAPLQHADQAVVVTANDFDSIYAMNRIIAAVQAKSKNYKVRLAGCVANRSRATDEVDRFCKETNFRRLAHMPDLDAIRRSRLKKKTLFEMDEDQDVLAARAEYIRLAESLWRGLDPI
DPHSLPDRDIFELLGFD

FFFFFFFFFFFFFFFFFFFFFFFFFFFFFXXXXXXXXXXXXXXXXXXXXXXXXXXXXXXXXXXXXXXXXXXXXXXXXXXXXXXXXXXXXXXXXXXXXXXXXXXXXXXXXXXXXXXXXXXXXXXXX
XXXXXXXXXXXXXXXXXXXXXXXXXXXXXXXXXXXXXXXXXXXXXXXXXXXXXXXXXXXXXXXXXXXXXXXXXXXXXXXXXXXXXXXXXXXXXXXXXXXXXXXXXXXXXXXXXXXXXXXXXXXXXXXXXXXXXXXX
XXXXXXXXXXXXXXXXX
>G4VQX9; DP03792
MDEFEEYRNPLTKRYASREMVCNFGEKRKVILWRQLWIWLAETQKELGFDITDEQINEMKSQRDSVDFGTAAAEEKARRHDVMAHVYTFALACPKAAPIIHLGATSCFVGDNADLIMLKDGLNILLPKVARCIDRLAKKA
MLHKSLICLARTHLQPAQPTTMGRRICMWIQDLLLDLENLERLKNHTIRFRGAKGAVGTQASFMDLFQGDHQKVIKLDEILTKKSGFQRSWCVTGQTYPRKVDIEITNALSNIGATVHKICTDIRLLSSFHEVEEPFETK
QIGSSAMPYKRNPIRSERACSLARYLMHISTSMVSTVSVQWLERSLDDSAIRRIVLPEAFLAADACLTLLQNIAEGLIVYPMVMEANLNSELPFLVVERILVKMVSEGAANRQECHERLRKHSHEAAAEIKLKGLKNSLM
DKLLNDYYFAPIHSLLPTVLDPSYMIGRAVEQVEVFLNTEVDPAIHSYKDCLALNSNITI
XXXXXXXXXXXXXXXXXXXXXXXXXXXXXXXXXXXXXXXXXXXXXXXXXXXXXXXXXXXXXXXXXXXXXXXXXXXXXXXXXXXXXXXXXXXXXXXXXXXXXXXXXXXXXXXXXXXXXXXXXXXXXXXXXXXXXXXX
XXXXXXXXXXXXXXXXXXXXXXXXXXXXXXXXXXXXXXXXXXXXXXXXXXXXXXXXXXXXXXXXXXXXXXXXXXXXXXXXXXXXXXXXXXXXXXXXXXXXXXXXXXXXXXXXXXXXXXXXXXXXXXXXXXXXXXXF
FFFFFFFFFXXXXXXXXXXXXXXXXXXXXXXXXXXXXXXXXXXXXXXXXXXXXXXXXXXXXXXXXXXXXXXXXXXXXXXXXXXXXXXXXXXXXXXXXXXXXXXXXXXXXXXXXXXXXXXXXXXXXXXXXXXXXX
XXXXXXXXXXXXXXXXXXXXXXXXXXXXXXXXXXXXXXXXXXXXXXXXXXXXXXXXXXXX
>P07598; DP00108
MSRTVMERIEYEMHTPDPKADPDKLHFVQIDEAKCIGCDTCSQYCPTAAIFGEMGEPHSIPHIEACINCGQCLTHCPENAIYEAQSWVPEVEKKLKDGKVKCIAMPAPAVRYALGDAFGMPVGSVTTGKMLAALQKLGFA
HCWDTEFTADVTIWEEGSEFVERLTKKSDMPLPQFTSCCPGWQKYAETYYPELLPHFSTCKSPIGMNGALAKTYGAERMKYDPKQVYTVSIMPCIAKKYEGLRPELKSSGMRDIDATLTTRELAYMIKKAGIDFAKLPDG
KRDSLMGESTGGATIFGVTGGVMEAALRFAYEAVTGKKPDSWDFKAVRGLDGIKEATVNVGGTDVKVAVVHGAKRFKQVCDDVKAGKSPYHFIEYMACPGGCVCGGGQPVMPGVLEAMDRTTTRLYAGLKKRLAMASANK
A
XXXXXXXXXXXXXXXXXXXXXXXXXXXXXXXXXXXXXXXXXXXXXXXXXXXXXXXXXXXXXXXXXXXXXXXXXXXXXXXXXXXXXXXXXXXXXXXXXXXXXXXXXXXXXXXXXXXXXXXXXXXXXXXXXXXXXXXX
XXXXXXXXXXXXXXXXXXXXXXXXXXXXXXXXXXXXXXXXXXXXXXXXXXXXXXXXXXXXXXXXXXXXXXXXXXXXXXXXXXXXXXXXXXXXXXXXXXXXXXXXXXXXXXXXXXXXXXXXXXXXXXXXXXXXXXXX
XXXXXXXXXXXXXXXXXXXXXXXXXXXXXXXXXXXXXXXXXXXXXXXXXXXXXXXXXXXXXXXXXXXXXXXXXXXXXXXXXXXXXXXXXXXXXXXXXXXXXXXXXXXXXXXXXXXXXXFFFFFFFFFFFFFFFFFFFFFF
F
>Q57WT9; DP04054
MGDDGYSSYVLLQEQILTVKRSFSEALEKELNLVEVRAPILFRVGDGTQDNLSGFEKAVQVPVKAIPNASFEVVHSLAKWKRRTLANYKFAPGHGLYTHMTALRVDDVLDNIHSVVVDQWDWEMVMKDDQRNLAFLKEVV
CKVYAAIRKTELAVCEKYKQKPILPETIQFVHAEHLLLAYPNLTAKEREREIAREYGAVFLIGIGAVLSSGDRHDARAPDYDDWTSPVEASQVVFPRTSKPIPTMNSLSSLKGLNGDILLYNPTLDDSLEVSSMGIRVNA
EALRHQISLTGDDSLLKSEWHQQLLNGEFPQTVGGGIGQSRMVMFMLRKKHIGEVQCSVWPEEIRKKHNLL
XXXXXXXXXXXXXXXXXXXXXXXXXXXXXXXXXXXXXXXXXXXXXXXXXXXXXXXXXXXXXXXXXXXXXXXXXXXXXXXXXXXXXXXXXXXXXXXXXXXXXXXXXXXXXXXXXXXXXXXXXXXXXXXXXXXXXXXX
XXXXXXXXXXXXXXXXXXXXXXXXXXXXXXXXXXXXXXXXXXXXXXXXXXXXXXXXXXXXXXXXXXXXXFFFFFFFFFFFFFFFFFFFFFFFFFFFFFFFFFFFFFXXXXXXXXXXXXXXXXXXXXXXXXXXXXXXXXXX
XXXXXXXXXXXXXXXXXXXXXXXXXXXXXXXXXXXXXXXXXXXXXXXXXXXXXXXXXXXXXXXXXXXXXXX
>P21575; DP02617
MGNRGMEDLIPLVNRLQDAFSAIGQNADLDLPQIAVVGGQSAGKSSVLENFVGRDFLPRGSGIVTRRPLVLQLVNSTTEYAEFLHCKGKKFTDFEEVRLEIEAETDRVTGTNKGISPVPINLRVYSPHVLNLTLVDLPGM
TKVPVGDQPPDIEFQIRDMLMQFVTKENCLILAVSPANSDLANSDALKIAKEVDPQGQRTIGVITKLDLMDEGTDARDVLENKLLPLRRGYIGVVNRSQKDIDGKKDITAALAAERKFFLSHPSYRHLADRMGTPYLQKV
LNQQLTNHIRDTLPGLRNKLQSQLLSIEKEVDEYKNFRPDDPARKTKALLQMVQQFAVDFEKRIEGSGDQIDTYELSGGARINRIFHERFPFELVKMEFDEKELRREISYAIKNIHGIRTGLFTPDLAFEATVKKQVQKL
KEPSIKCVDMVVSELTSTIRKCSEKLQQYPRLREEMERIVTTHIREREGRTKEQVMLLIDIELAYMNTNHEDFIGFANAQQRSNQMNKKKTSGNQDEILVIRKGWLTINNIGIMKGGSKEYWFVLTAENLSWYKDDEEKE
KKYMLSVDNLKLRDVEKGFMSSKHIFALFNTEQRNVYKDYRQLELACETQEEVDSWKASFLRAGVYPERVGDKEKASETEENGSDSFMHSMDPQLERQVETIRNLVDSYMAIVNKTVRDLMPKTIMHLMINNTKEFIFSE
LLANLYSCGDQNTLMEESAEQAQRRDEMLRMYHALKEALSIIGDINTTTVSTPMPPPVDDSWLQVQSVPAGRRSPTSSPTPQRRAPAVPPARPGSRGPAPGPPPAGSALGGAPPVPSRPGASPDPFGPPPQVPSRPNRAP
PGVPSRSGQASPSRPESPRPPFDL
XXXXXXXXXXXXXXXXXXXXXXXXXXXXXXXXXXXXXXXXXXXXXXXXXXXXXXXXXXXXXXXXXXXXXXXXXXXXXXXXXXXXXXXXXXXXXXXXXXXXXXXXXXXXXXXXXXXXXXXXXXXXXXXXXXXXXXXX
XXXXXXXXXXXXXXXXXXXXXXXXXXXXXXXXXXXXXXXXXXXXXXXXXXXXXXXXXXXXXXXXXXXXXXXXXXXXXXXXXXXXXXXXXXXXXXXXXXXXXXXXXXXXXXXXXXXXXXXXXXXXXXXXXXXXXXXX
XXXXXXXXXXXXXXXXXXXXXXXXXXXXXXXXXXXXXXXXXXXXXXXXXXXXXXXXXXXXXXXXXXXXXXXXXXXXXXXXXXXXXXXXXXXXXXXXXXXXXXXXXXXXXXXXXXXXXXXXXXXXXXXXXXXXXXXX
XXXXXXXXXXXXXXXXXXXXXXXXXXXXXXXXXXXXXXXXXXXXXXXXXXXXXXXXXXXXXXXXXXXXXXXXXXXXFFFFFFFFFFFFFFFFFFFFFXXXXXXXXXXXXXXXXXXXXXXXXXXXXXXXXXXXXXXXXXXX
XXXXXXXXXXXXXXXXXXXXXXXXXXXXXXXXXXXXXXXXXXXXXXXXXXXXXXXXXXXXXXXXXXXXFFFFFFFFFFFFFFFFFFFFFFFFXXXXXXXXXXXXXXXXXXXXXXXXXXXXXXXXXXXXXXXXXXXXXXXX
XXXXXXXXXXXXXXXXXXXXXXXXXXXXXXXXXXXXXXXXXXXXXXXXXXXXXXXXXXXXXXXXXXXXXXXXXXXXXXXXXXXXXXXXXXXXXXXXXXXXXXXXXXXXXXXXXXXXXXXXXXXXXXXXXXXXXXXX
XXXXXXXXXXXXXXXXXXXXXXXX
>Q9JYY3; DP03579
MSNENRTCSFCGKSKSHVKHLIEGENAFICDECVSNCIEILHEDGNDGTPSESAGGEPEESGKLPTPAEIVANLNDHVIGQEQAKKALAVSVYNHYKRLRHPKAGANVELSKSNILLIGPTGSGKTLLAQSLARKLDVPF
VMADATTLTEAGYVGEDVEQIITKLLGKCDFDVEKAQRGIVYIDEIDKISRKSDNPSITRDVSGEGVQQALLKLIEGTVASVPPQGGRKHPNQEFINVDTTNILFICGGAFAGLEKVIRQRTEKGGIGFGASVHSKDENA
DITKLFGIVEPEDLIKFGLIPELIGRLPVIATLEELDEDALINILTEPKNALVKQYQALFGMENVELEFEEGALRSIARQAMERKTGARGLRSIVERCLLDTMYRLPDLKGLKKVVVGKAVIEEGREPELVFES
FFFFFFFFFFFFFFFFFFFFFFFFFFFFFFFFFFFFFFFFFFFFFFFFFFFFFFFFFFFFFFXXXXXXXXXXXXXXXXXXXXXXXXXXXXXXXXXXXXXXXXXXXXXXXXXXXXXXXXXXXXXXXXXXXXXXXXXXXXXX
XXXXXXXXXXXXXXXXXXXXXXXXXXXXXXXXXXXXXXXXXXXXXXXXXXXXXXXXXXXXXXXXXXXXXXXXXXXXXXXXXXXXXXXXXXXXXXXXXXXXXXXXXXXXXXXXXXXXXXXXXXXXXFFFFFFFFFFFFFFF
XXXXXXXXXXXXXXXXXXXXXXXXXXXXXXXXXXXXXXXXXXXXXXXXXXXXXXXXXXXXXXXXXXXXXXXXXXXXXXXXXXXXXXXXXXXXXXXXXXXXXXXXXXXXXXXXXXXXXXXXXXXXXXXXXXXXXX
>P07363; DP00407
MSMDISDFYQTFFDEADELLADMEQHLLVLQPEAPDAEQLNAIFRAAHSIKGGAGTFGFSVLQETTHLMENLLDEARRGEMQLNTDIINLFLETKDIMQEQLDAYKQSQEPDAASFDYICQALRQLALEAKGETPSAVTR
LSVVAKSEPQDEQSRSQSPRRIILSRLKAGEVDLLEEELGHLTTLTDVVKGADSLSAILPGDIAEDDITAVLCFVIEADQITFETVEVSPKISTPPVLKLAAEQAPTGRVEREKTTRSNESTSIRVAVEKVDQLINLVGE
LVITQSMLAQRSSELDPVNHGDLITSMGQLQRNARDLQESVMSIRMMPMEYVFSRYPRLVRDLAGKLGKQVELTLVGSSTELDKSLIERIIDPLTHLVRNSLDHGIELPEKRLAAGKNSVGNLILSAEHQGGNICIEVTD

DGAGLNRERILAKAASQGLTVSENMSDDEVAMLIFAPGFSTAEQVTDVSGRGVGMDVVKRNIQKMGGHVEIQSKQGTGTTIRILLPLTLAILDGMSVRVADEVFILPLNAVMESLQPREADLHPLAGGERVLEVRGEYLP
IVELWKVFNVAGAKTEATQGIVVILQSGGRRYALLVDQLIGQHQVVVKNLESNYRKVPGISAATILGDGSVALIVDVSALQAINREQRMANTAA
XXXXXXXXXXXXXXXXXXXXXXXXXXXXXXXXXXXXXXXXXXXXXXXXXXXXXXXXXXXXXXXXXXXXXXXXXXXXXXXXXXXXXXXXXXXXXXXXXXXXXXXXXXXXXXXXXXXXXXXXXFFFFFFFFFFFFFFFFF
FFFFFFFFFFFFFFFFFFXXXXXXXXXXXXXXXXXXXXXXXXXXXXXXXXXXXXXXXXXXXXXXXXXXXXXFFFFFFFFFFFFFFFFFFFFFFFFFFFFFFXXXXXXXXXXXXXXXXXXXXXXX
XXXXXXXXXXXXXXXXXXXXXXXXXXXXXXXXXXXXXXXXXXXXXXXXXXXXXXXXXXXXXXXXXXXXXXXXXXXXXXXXXXXXXXXXXXXXXXXXXXXXXXXXXXXXXXXXXXXXXXXXXXXXXXXXXXXXXXXXXX
XXXXXXXXXXXXXXXXXXXXXXXXXXXXXXXXXXXXXXXXXXXXXXXXXXXXXXXXXXXXXXXXXXXXXXXXXXXXXXXXXXXXXXXXXXXXXXXXXXXXXXXXXXXXXXXXXXXXXXXXXXXXXXXXXXXXXXXXXX
XXXXXXXXXXXXXXXXXXXXXXXXXXXXXXXXXXXXXXXXXXXXXXXXXXXXXXXXXXXXXXXXXXXXXXXXXXXXXXXXXXXXXXXXXXXXXX
>P41214; DP02704
MFAKAFRVKSNTAIKGSDRRKLRADVTTAFPTLGTDQVSELVPGKEELNIVKLYAHKGDAVTVYVSGGNPILFELEKNLYPTVYTLWSYPDLLPTFTTWPLVLEKLVGGADLMLPGLVMPPAGLPQVQKGDLCAISLVGN
RAPVAIGVAAMSTAEMLTSGLKGRGFSVLHTYQDHLWRSGNKSSPPSIAPLALDSADLSEEKGSVQMDSTLQGDMRHMTLEGEEENGEVHQAREDKSLSEAPEDTSTRGLNQDSTDSKTLQEQMDELLQQCFLHALKCRV
KKADLPLLTSTFLGSHMFSCCPEGRQLDIKKSSYKKLSKFLQQMQQEQIIQVKELSKGVESIVAVDWKHPRITSFVIPEPSPTSQTIQEGSREQPYHPPDIKPLYCVPASMTLLFQESGHKKGSFLEGSEVRTIVINYAK
KNDLVDADNKNLVRLDPILCDCILEKNEQHTVMKLPWDSLLTRCLEKLQPAYQVTLPGQEPIVKKGRICPIDITLAQRASNKKVTVVRNLEAYGLDPYSVAAILQQRCQASTTVNPAPGAKDSLQVQIQGNQVHHLGWLL
LEEYQLPRKHIQGLEKALKPGKKK
XXXXXXXXXXXXXXXXXXXXXXXXXXXXXXXXXXXXXXXXXXXXXXXXXXXXXXXXXXXXXXXXXXXXXXXXXXXXXXXXXXXXXXXXXXXXXXXXXXXXXXXXXXXXXXXXXXXXXXXXXXXXXXXXXXXXXXXXXX
XXXXXXXXXXXXXXXXXXXXXXXXXXXXXXXXXXXXXXXXXXXFFFFFFFFFFFFFFFFFFFFFFFFFFFFFFFFFFFFFFFFFFFFFFFFFFFFFFFFFFFFFFFFFFFFFFFFFFFFFFFFFFFXXXXXXXXXXXXXXXXXXXXX
XXXXXXXXXXXXXXXXXXXXXXXXXXXXXXXXXXXXXXXXXXXXXXXXXXXXXXXXXXXXXXXXXXXXXFFFFFFFFFFFFFFFFFFFFFFFFFFXXXXXXXXXXXXXXXXXXXXXXXXXXXXXXXXXXXXXXXXXXXXXXX
XXXXXXXXXXXXXXXXXXXXXXXXXXXXXXXXXXXXXXXXXXXXXXXXXXXXXXXXXXXXXXXXXXXXXXXXXXXXXXXXXXXXXXXXXXXXXXXXXXXXXXXXXXXXXXXXXXXXXXXXXXXXXXXXXXXXXXXXXX
XXXXXXXXXXXXXXXXXXXXXXXX
>P40484; DP03995
MSFLQNFHISPGQTIRSTRGFKWNTANAANNAGSVSPTKATPHNNTINGNNNNANTINNRADFTNNPVNGYNESDHGRMSPVLTTPKRHAPPPEQLQNVTDFNYTPSHQKPFLQPQAGTTVTTHQDIKQIVEMTLGSEGV
LNQAVKLPRGEDENEWLAVHCVDFYNQINMLYGSITEFCSPQTCPRMIATNEYEYLWAFQKGQPPVSVSAPKYVECLMRWCQDQFDDESLFPSKVTGTFPEGFIQRVIQPILRRLFRVYAHIYCHHFNEILELNLQTVLN
TSFRHFCLFAQEFELLRPADFGPLLELVMELRDR
XXXXXXXXXXXXXXXXXXXXXXXXXXXXXXXXXXXXXXXXXXXXXXXXXXXXXXXXXXXXXXXXXXXXXXXXXXXXXXXXXXXXXFFFFFFFFFFFFXXXXXXXXXXXXXXXXFFFFFFFFFFFFXXXXXXXXXXXXXXX
XXXXXXXXXXXXXXXXXXXXXXXXXXXXXXXXXXXXXXXXXXXXXXXXXXXXXXXXXXXXXXXXXXXXXXXXXXXXXXXXXXXXXXXXXXXXXXXXXXXXXXXXXXXXXXXXXXXXXXXXXXXXXXXXXXXXXXXXXX
XXXXXXXXXXXXXXXXXXXXXXXXXXXXXXXXXX
>O08908; DP02791
MAGAEGFQYRAVYPFRRERPEDLELLPGDLLVVSRVALQALGVADGGERCPHNVGWMPGFNERTRQRGDFPGTYVEFLGPVALARPGPRPRGPRPLPARPLDGSSESGHILPDLAEQFSPPDPAPPILVKLVEAIEQAEL
DSECYSKPELPATRTDWSLSDLEQWDRTALYDAVKGFLLALPAAVVTPEAAAEAYRALREVAGPVGLVLEPPTLPLHQALTLRFLLQHLGRVARRAPSPDTAVHALASAFGPLLLRIPPSGGEGDGSEPVPDFPVLLLER
LVQEHVEEQDAAPPALPPKPSKAKPAPTALANGGSPPSLQDAEWYWGDISREEVNERLRDTPDGTFLVRDASSKIQGEYTLTLRKGGNNKLIKVFHRDGHYGFSEPLTFCSVVELISHYRHESLAQYNAKLDTRLLYPVS
KYQQDQVVKEDSIEAVGAQLKVYHQQYQDKSREYDQLYEEYTRTSQELQMKRTAIEAFNETIKIFEEQGQTQEKCSKEYLERFRREGNEKEMQRILLNSERLKSRIAEIHESRTKLEQDLRAQASDNREIDKRMNSLKPD
LMQLRKIRDQYLVWLTQKGARQRKINEWLGIKNETEDQYSLMEDEDALPHHEERTWYVGKINRTQAEEMLSGKRDGTFLIRESSQRGCYACSVVVDGDTKHCVIYRTATGFGFAEPYNLYGSLKELVLHYQHASLVQHND
ALTVTLAHPVRAPGPGPPSAAR
XXXXXXXXXXXXXXXXXXXXXXXXXXXXXXXXXXXXXXXXXXXXXXXXXXXXXXXXXXXXXXXXXXXXXXXXXXXXXXXXXXXXXXXXXXXXXXXXXXXXXXXXXXXXXXXXXXXXXXXXXXXXXXXXXXXXXXXXXX
XXXXXXXXXXXXXXXXXXXXXXXXXXXXXXXXXXXXXXXXXXXXXXXXXXXXXXXXXXXXXXXXXXXXXXXXXXXXXXXXXXXXXXXXXXXXXXXXXXXXXXXXXXXXXXXXXXXXXXXXXXXXXXXXXXXXXXXXX
XXXXXXXXXXXXXXXXXXXXXXXXXXXXXXXXXXXXXXXXXXXXXXXXXXXXXXXXXXXXXXXXXXXXXXXXXXXXXXXXXXXXXXXXXXXXXXXXXXXXXXXXXXXXXXXXXXXXXXXXXXXXXXXXXXXXXXXXXX
XXFFFFFFFFFFFFFFFFFFXXXXXXXXXXXXXXXXXXXXXXXXXXXXXXXXXXXXXXXXXXXXXXXXXXXXXXXXXXXXXXXXXXXXXXXXXXXXXXXXXXXXXXXXXXXXXXXXXXXXXXXXXXXXXXXXXXXXXXXX
XXXXXXXXXXXXXXXFFFFFFFFFFFFFFFFFFFFFFFFFFFFFFFFXXXXXXXXXXXXXXXXXXXXXXXXXXXXXXXXXXXXXXXXXXXXXXXXXXXXXXXXXXXXXXXXXXXXXXXXXXXXXXXXXXXXXXXXXXXXXX
XXXXXXXXXXXXXXXXXXXXXX
>Q07412; DP00614
MARKYFVAANWKCNGTLESIKSLTNSFNNLDFDPSKLDVVVFPVSVHYDHTRKLLQSKFSTGIQNVSKFGNGSYTGEVSAEIAKDLNIEYVIIGHFERRKYFHETDEDVREKLQASLKNNLKAVVCFGESLEQREQNKTI
EVITKQVKAFVDLIDNFDNVILAYEPLWAIGTGKTATPEQAQLVHKEIRKIVKDTCGEKQANQIRILYGGSVNTENCSSLIQQEDIDGFLVGNASLKESFVDIIKSAM
XXXXXXXXXXXXXXXXXXXXXXXXXXXXXXXXXXXXXXXXXXXXXXXXXXXXXXXXXXXXXXXXXFFFFFFFFFFFFFFFXXXXXXXXXXXXXXXXXXXXXXXXXXXXXXXXXXXXXXXXXXXXXXXXXXXXXXXXXXXXX
XXXXXXXXXXXXXXXXXXXXXXXXFFFFFFFFFFFFFFXXXXXXXXXXXXXXXXXXXXXXXXXXXXXXXXXXXXXXXXXXXXXXXXXXXXXXXXXXXXXXXXXXXXXX
>Q95V77; DP00186
MSSQQNQNRQGEQQEQGYMEAAKEKVVNAWESTKETLSSTAQAAAEKTAEFRDSAGETIRDLTGQAQEKGQEFKERAGEKAEETKQRAGEKMDETKQRAGEMRENAGQKMEEYKQQGKGKAEELRDTAAEKLHQAGEKVK
GRD
FFFFFFFFFFFFFFFFFFFFFFFFFFFFFFFFFFFFFFFFFFFFFFFFFFFFFFFFFFFFFFFFFFFFFFFFFFFFFFFFFFFFFFFFFFFFFFFFFFFFFFFFFFFFFFFFFFFFFFFFFFFFFFFFFFFFFFFFFFFF
FFF
>P26645; DP00253
MGAQFSKTAAKGEATAERPGEAAVASSPSKANGQENGHVKVNGDASPAAAEPGAKEELQANGSAPAADKEEPASGSAATPAAAEKDEAAAATEPGAGAADKEAAEAEPAEPSSPAAEAEGASASSTSSPKAEDGAAPSPS
SETPKKKKKRFSFKKSFKLSGFSFKKSKKESGEGAEAEGATAEGAKDEAAAAAGGEGAAAPGEQAGGAGAEGAAGGEPREAEAAEPEQPEQPEQPAAEEPQAEEQSEAAGEKAEEPAPGATAGDASSAAGPEQEAPAATD
EAAASAAPAASPEPQPECSPEAPPAPTAE

FFFFFFFFFFFFFFFFFFFFFFFFFFFFFFFFFFFFFFFFFFFFFFFFFFFFFFFFFFFFFFFFFFFFFFFFFFFFFFFFFFFFFFFFFFFFFFFFFFFFFFFFFFFFFFFFFFFFFFFFFFFFFFFFFFFFFFFFFFFF
FFFFFFFFFFFFFFFFFFFFFFFFFFFFFFFFFFFFFFFFFFFFFFFFFFFFFFFFFFFFFFFFFFFFFFFFFFFFFFFFFFFFFFFFFFFFFFFFFFFFFFFFFFFFFFFFFFFFFFFFFFFFFFFFFFFFFFFFFFFF
FFFFFFFFFFFFFFFFFFFFFFFFFFFFF
>P38936; DP00016
MSEPAGDVRQNPCGSKACRRLFGPVDSEQLSRDCDALMAGCIQEARERWNFDFVTETPLEGDFAWERVRGLGLPKLYLPTGPRRGRDELGGGRRPGTSPALLQGTAEEDHVDLSLSCTLVPRSGEQAEGSPGGPGDSQGR
KRRQTSMTDFYHSKRRLIFSKRKP
FFFFFFFFFFFFFFFFFFFFFFFFFFFFFFFFFFFFFFFFFFFFFFFFFFFFFFFFFFFFFFFFFFFFFFFFFFFFFFFFFFFFFFFFFFFFFFFFFFFFFFFFFFFFFFFFFFFFFFFFFFFFFFFFFFFFFFFFFFFF
FFFFFFFFFFFFFFFFFFFFFFFF
>A2VD23; DP00584
MLSRTVFVPLILAFVGVSISAPVSNNNGTDNDESAADQRHIFTVQFNVGTPAPADGDSVTTDGKDSAEKNEAPGDSSDTTEKPGTTDGKDSAEQHGVTTDGKDEAEQHGVTTDGQDSAEKRGEADGAPDKPDTQNGTDDT
DSDQETDASHHKTGDSDENKDKPSAEDHTDGNHAGKDSTDSKESPDTTDKPEGPDSDSAPDGDSASAEKTDSDHSPDEDANKSSTEADKDDTSDKDSSQTDEKHDSDASDKDEKHEDKDEKSDEKDSSKDSEDKSQEKSD
KSDDGSNSEADEQKESVESKDHDSDSQDSDSAEKKEKHDDKDQDSSDSADSKDSDEDKDKDHSEQKDSEDHEHKEKHTKDKEEHKDSDEGKDDEDKSKSDEHDKDESESKEASKSDESEQEEKKDDKSDSDNSSRDSHSD
SDSDSHSDSDSDSHSDSHSDSDSDSHSDSDSDSDSDSDSDSDSDSDSNSRDKDEKKDKSSESRDEDSSDSDSKSNSESSETAEEDTNDDKDSSVEKDKTDSSDSASVEANDSDDEHDDDSKDATPSSEDHTAEKTDEDSH
DVSDDDDDIDAHDDEAGVEHGTDEASKPHQEPDHHDDTTHGSDDGRKTSMPIS
FFFFFFFFFFFFFFFFFFFFFFFFFFFFFFFFFFFFFFFFFFFFFFFFFFFFFFFFFFFFFFFFFFFFFFFFFFFFFFFFFFFFFFFFFFFFFFFFFFFFFFFFFFFFFFFFFFFFFFFFFFFFFFFFFFFFFFFFFFFF
FFFFFFFFFFFFFFFFFFFFFFFFFFFFFFFFFFFFFFFFFFFFFFFFFFFFFFFFFFFFFFFFFFFFFFFFFFFFFFFFFFFFFFFFFFFFFFFFFFFFFFFFFFFFFFFFFFFFFFFFFFFFFFFFFFFFFFFFFFFF
FFFFFFFFFFFFFFFFFFFFFFFFFFFFFFFFFFFFFFFFFFFFFFFFFFFFFFFFFFFFFFFFFFFFFFFFFFFFFFFFFFFFFFFFFFFFFFFFFFFFFFFFFFFFFFFFFFFFFFFFFFFFFFFFFFFFFFFFFFFF
FFFFFFFFFFFFFFFFFFFFFFFFFFFFFFFFFFFFFFFFFFFFFFFFFFFFFFFFFFFFFFFFFFFFFFFFFFFFFFFFFFFFFFFFFFFFFFFFFFFFFFFFFFFFFFFFFFFFFFFFFFFFFFFFFFFFFFFFFFFF
FFFFFFFFFFFFFFFFFFFFFFFFFFFFFFFFFFFFFFFFFFFFFFFFFFFFF
>O95997; DP00521
MATLIYVDKENGEPGTRVVAKDGLKLGSGPSIKALDGRSQVSTPRFGKTFDAPPALPKATRKALGTVNRATEKSVKTKGPLKQKQPSFSAKKMTEKTVKAKSSVPASDDAYPEIEKFFPFNPLDFESFDLPEEHQIAHLP
LSGVPLMILDEERELEKLFQLGPPSPVKMPSPPWESNLLQSPSSILSTLDVELPPVCCDIDI
FFFFFFFFFFFFFFFFFFFFFFFFFFFFFFFFFFFFFFFFFFFFFFFFFFFFFFFFFFFFFFFFFFFFFFFFFFFFFFFFFFFFFFFFFFFFFFFFFFFFFFFFFFFFFFFFFFFFFFFFFFFFFFFFFFFFFFFFFFFF
FFFFFFFFFFFFFFFFFFFFFFFFFFFFFFFFFFFFFFFFFFFFFFFFFFFFFFFFFFFFFF
>Q7MXL4
MKPTLFVLAAGMGSRYGSLKQLDGIGPGGDTIMDYSVYDAIRAGFGRLVFVIRHSFEKEFREKILTKYEGRIPVELVFQELDRLPEGFSCPEGREKPWGTNHAVLMGRDAIREPFAVINADDFYGRNGFEVLARKLMTLE
GKQGEYCMVGYRVGNTLSESGGVSRGVCQVDEKHLLTGVVERTGIERTDGTISFRDETGKICTLAEDAPVSMNMWGFTPDYFDYSEELFINFLNAHGQEPKSEFFIPFVVNDLIRSGRASVEVLDTTARWFGVTYSDDRP
GVVAKLRELTEAGEYPTKLF
SSSSSSSSSSSSSSSSSSSSSSSSSSSSSSSSSSSSSSSSSSSSSSSSSSSSSSSSSSSSSSSSSSSSSSSSSSSSSSSSSSSSSSSSSSSSSSSSSSSSSSSSSSSSSSSSSSSSSSSSSSSSSSSSSSSSSSSSS
SSSSSSSSSSSSSSSSSSSSSSSSSSSSSSSSSSSSSSSSSSSSSSSSSSSSSSSSSSSSSSSSSSSSSSSSSSSSSSSSSSSSSSSSSSSSSSSSSSSSSSSSSSSSSSSSSSSSSSSSSSSSSSSSSSSSSSSS
SSSSSSSSSSSSSSSSSSSS
>Q9RUY4
MSKMDQLRPVLGRAGVDALWVSAPANVRWLSGFTSAEDGKVLVSPDGATLYTDARYTVQAQEESSLPQYIARPPATYEHAADTVRGLRVGFEAESLTVAELEDLRQAWPNSTLVALRGTLGGLRAVKTPEEIGAIRAAQD
LADRVYTEVRPMIRAGVRELDVAVEIETRLRRAGGESAFELIVASGPNGAKPHGHASKRVIEDGDLVTIDMGARLGGYNSDMTRTVAVGTPSAEMKRVYDAVLEAEEAAIAAIRPGVRAADLDKLARDLLTRHGLGEAFA
HSLGHGVGLEVHEGPGLRGTSQDVLEAGMVITIEPGAYLPGVGGVRIEDLILVTEDGYEVLSHSAKESV
SSSSSSSSSSSSSSSSSSSSSSSSSSSSSSSSSSSSSSSSSSSSSSSSSSSSSSSSSSSSSSSSSSSSSSSSSSSSSSSSSSSSSSSSSSSSSSSSSSSSSSSSSSSSSSSSSSSSSSSSSSSSSSSSSSSSSSSSS
SSSSSSSSSSSSSSSSSSSSSSSSSSSSSSSSSSSSSSSSSSSSSSSSSSSSSSSSSSSSSSSSSSSSSSSSSSSSSSSSSSSSSSSSSSSSSSSSSSSSSSSSSSSSSSSSSSSSSSSSSSSSSSSSSSSSSSSS
SSSSSSSSSSSSSSSSSSSSSSSSSSSSSSSSSSSSSSSSSSSSSSSSSSSSSSSSSSSSSSSSSSSSS
>Q2YQ74
MTSPRNCLRFTLLGCGSSPGVPRINGDWGKCDPKNPKNRRRRASLLVERYDAEGNNTVVVIDTGPDFRMQMIDSGVHMLDAAVYTHPHADHIHGIDDLRTYVVDNGRLMDVYANRLTRNRLYDTFGYCFETPVGSSYPPI
LSMHDIAPETPFSIEGAGGAIRFEPFSQVHGDIESLGFRIGSVVYCTDVSAFPEQSLQYIKDADVLIIGALQYRPHPSHFSLGEALEWIEKLSPKRAILTHMHVPLDYETVMRETPHHVEPGYDGLRFEVAV
SSSSSSSSSSSSSSSSSSSSSSSSSSSSSSSSSSSSSSSSSSSSSSSSSSSSSSSSSSSSSSSSSSSSSSSSSSSSSSSSSSSSSSSSSSSSSSSSSSSSSSSSSSSSSSSSSSSSSSSSSSSSSSSSSSSSSSSSSS
SSSSSSSSSSSSSSSSSSSSSSSSSSSSSSSSSSSSSSSSSSSSSSSSSSSSSSSSSSSSSSSSSSSSSSSSSSSSSSSSSSSSSSSSSSSSSSSSSSSSSSSSSSSSSSSSSSSSSSSSSSSSSSSS
>P55342
MQLTELSIKNQNVFVQHYIDGKEEMSSFFDYSIHHKDMWRERLEDLSSRFFAREELAAYLTSYHNKFGSSAMQSAIEKLKDPSSAAVVGGQQAGLLTGPLYTIHKIISIIVLAKQQEKELQVPVIPIFWVAGEDHDLDEI
NFVHTSEENGPVKKKLPQSYWKKSSAASTSLDQEKCAAWIDDVFAAFEETDHTNTLLDNVKRCLRESVTFTDFFELLIADLFQEEGLVLLNSGDPGIKKLETAMFQKILRENDELARAVSDQQAFMRQAGYKPIIESGKE
QANLFYEYEDERFLIEKDNGRFVIKELDLGWTRDELHTHMEEHPERFSNNVVTRPLMQEFLIPTLAFIAGPGEINYWGELKQAFAVMGFKMPPVMPRLNITILERHIEKKLAERNISLQDAIERGTENQRETYFERQIPE
EFTAVMDQAKSQIEAIHKTVRQEALKVDQSLEPLLLKNAAFIQDQLQFLERTVMKRIEEKEGYVLKDYERIQNSIKPLLAPQERIWNIMYYLNRYGPKFFTTFKNLPFSFQNQHQVVKL
SSSSSSSSSSSSSSSSSSSSSSSSSSSSSSSSSSSSSSSSSSSSSSSSSSSSSSSSSSSSSSSSSSSSSSSSSSSSSSSSSSSSSSSSSSSSSSSSSSSSSSSSSSSSSSSSSSSSSSSSSSSSSSSSSSSSSSSSSS
SSSSSSSSSSSSSSSSSSSSSSSSSSSSSSSSSSSSSSSSSSSSSSSSSSSSSSSSSSSSSSSSSSSSSSSSSSSSSSSSSSSSSSSSSSSSSSSSSSSSSSSSSSSSSSSSSSSSSSSSSSSSSSSSSSSSSSSS
SSSSSSSSSSSSSSSSSSSSSSSSSSSSSSSSSSSSSSSSSSSSSSSSSSSSSSSSSSSSSSSSSSSSSSSSSSSSSSSSSSSSSSSSSSSSSSSSSSSSSSSSSSSSSSSSSSSSSSSSSSSSSSSSSSSSSSSSSS
SSSSSSSSSSSSSSSSSSSSSSSSSSSSSSSSSSSSSSSSSSSSSSSSSSSSSSSSSSSSSSSSSSSSSSSSSSSSSSSSSSSSSSSSSSSSSSSSSSSSSSSSSSSSSSSSSSSSS
>P10537

MAPIPGVMPIGNYVSLYVMLPLGVVNADNVFPDKEKVEDELKQVKAGGCDGVMVDVWWGIIEAKGPKQYDWSAYRELFQLVKKCGLKIQAIMSFHQCGGNVGDAVFIPIPQWILQIGDKNPDIFYTNRAGNRNQEYLSLG
VDNQRLFQGRTALEMYRDFMESFRDNMADFLKAGDIVDIEVGCGAAGELRYPSYPETQGWVFPGIGEFQCYDKYMVADWKEAVKQAGNADWEMPGKGAGTYNDTPDKTEFFRPNGTYKTDMGKFFLTWYSNKLIIHGDQV
LEEANKVFVGLRVNIAAKVSGIHWWYNHVSHAAELTAGFYNVAGRDGYRPIARMLARHHATLNFTCLEMRDSEQPAEAKSAPQELVQQVLSSGWKEYIDVAGENALPRYDATAYNQMLLNVRPNGVNLNGPPKLKMSGLT
YLRLSDDLLQTDNFELFKKFVKKMHADLDPSPNAISPAVLERSNSAITIDELMEATKGSRPFPWYDVTDMPVDGSNPFD
SSSSSSSSSSSSSSSSSSSSSSSSSSSSSSSSSSSSSSSSSSSSSSSSSSSSSSSSSSSSSSSSSSSSSSSSSSSSSSSSSSSSSSSSSSSSSSSSSSSSSSSSSSSSSSSSSSSSSSSSSSSSSSSSSSSSSSSSSS
SSSSSSSSSSSSSSSSSSSSSSSSSSSSSSSSSSSSSSSSSSSSSSSSSSSSSSSSSSSSSSSSSSSSSSSSSSSSSSSSSSSSSSSSSSSSSSSSSSSSSSSSSSSSSSSSSSSSSSSSSSSSSSSSSSSSSSSS
SSSSSSSSSSSSSSSSSSSSSSSSSSSSSSSSSSSSSSSSSSSSSSSSSSSSSSSSSSSSSSSSSSSSSSSSSSSSSSSSSSSSSSSSSSSSSSSSSSSSSSSSSSSSSSSSSSSSSSSSSSSSSSSSSSSSSSSSSS
SSSSSSSSSSSSSSSSSSSSSSSSSSSSSSSSSSSSSSSSSSSSSSSSSSSSSSSSSSSSSSSSSSSSSSSSSSSSSSS
>A6TB44
MKVWPVKHSPLLRQPERFIARSELQALIRNVTQNLVNIKDESGQFLLRLDDGRVIDTKGWAGWEWTHGVGLYGIYQYYQQTGDIEMRDIIDRWFADRFAEGATTKNVNTMAPFLTLAYRFEETGRMAYLPWLESWAEWAM
HEMPRTEQGGMQHMTLAEENHQQMWDDTLMMTVLPLAKIGKLLNRPQYVEEATYQFLLHVQNLMDRETGLWFHGWNYEGRHNFARARWARGNSWLTMVIPDFLELVDLPEGNAVRRYLITVLDAQIAALAECQDDSGLWH
TLLDDPHSYLEASATAGFAYGILKAVRKRYVGQHYAGVAEKAIRGIVQNISPQGELLQTSFGTGMGSDLDFYRQIPLTSMPYGQAMAILCLTEYLRKYF
SSSSSSSSSSSSSSSSSSSSSSSSSSSSSSSSSSSSSSSSSSSSSSSSSSSSSSSSSSSSSSSSSSSSSSSSSSSSSSSSSSSSSSSSSSSSSSSSSSSSSSSSSSSSSSSSSSSSSSSSSSSSSSSSSSSSSSSSSS
SSSSSSSSSSSSSSSSSSSSSSSSSSSSSSSSSSSSSSSSSSSSSSSSSSSSSSSSSSSSSSSSSSSSSSSSSSSSSSSSSSSSSSSSSSSSSSSSSSSSSSSSSSSSSSSSSSSSSSSSSSSSSSSSSSSSSSSS
SSSSSSSSSSSSSSSSSSSSSSSSSSSSSSSSSSSSSSSSSSSSSSSSSSSSSSSSSSSSSSSSSSSSSSSSSSSSSSSSSSSSSSSSSSSSSSS
>Q1LBR9
MLSREDNELLVRVGPGTAMGTMMRLYWIPFLKSSEVTAGGQPYRVRLLGEDLVAFRDNSGNVGLVDHTCPHRGAPMVFGRNENDGLRCVYHGWKFKVSGQCEEMPCEPADSPMCKRMKIKAYPVKERNGILWAYMGPDAE
NAPELPDVEWNMVPEEQVAISMRVQECNWLQALEGELDSAHAAILHGRVGEGGVINQWRQAQDLSPTFECVQHDAGISIGARRKTPDGENYVRVNQFLMPFWTLVPPQSQFPELSGHAWVPIDDEHTLCLMFSYHPAKPF
YERTRKLFKEGHNGRETGHHSDNAFEKRPVTEPYHTYWSKFNRGNAYQFDYQSQVEKYNSGMPGLWIQDAACQSGTTPILDRSKEHLGTSDTGVARMRRVLLEAVKKLVATGEHPVSSNAPAAFRWRAVSLTIPLGGDWT
KLGEEAMRAEPGKDFGYTP
SSSSSSSSSSSSSSSSSSSSSSSSSSSSSSSSSSSSSSSSSSSSSSSSSSSSSSSSSSSSSSSSSSSSSSSSSSSSSSSSSSSSSSSSSSSSSSSSSSSSSSSSSSSSSSSSSSSSSSSSSSSSSSSSSSSSSSSS
SSSSSSSSSSSSSSSSSSSSSSSSSSSSSSSSSSSSSSSSSSSSSSSSSSSSSSSSSSSSSSSSSSSSSSSSSSSSSSSSSSSSSSSSSSSSSSSSSSSSSSSSSSSSSSSSSSSSSSSSSSSSSSSSSSSSSSSS
SSSSSSSSSSSSSSSSSSSSSSSSSSSSSSSSSSSSSSSSSSSSSSSSSSSSSSSSSSSSSSSSSSSSSSSSSSSSSSSSSSSSSSSSSSSSSSSSSSSSSSSSSSSSSSSSSSSSSSSSSSSSSSSSSSSSSSSS
SSSSSSSSSSSSSSSSSSS
>B1YEL6
MDVTIERVNDFDGYNWLPLLAKSSQEGFQLVERMLRNRREESFQEDGEAMFVALSTTNQVLACGGYMKQSGQARTGRIRHVYVLPEARSHGIGTALLEKIMSEAFLTYDRLVLYSEQADPFYQGLGFQLVSGEKITHTLD
KTAFADSNR
SSSSSSSSSSSSSSSSSSSSSSSSSSSSSSSSSSSSSSSSSSSSSSSSSSSSSSSSSSSSSSSSSSSSSSSSSSSSSSSSSSSSSSSSSSSSSSSSSSSSSSSSSSSSSSSSSSSSSSSSSSSSSSSSSSSSSSSS
SSSSSSSSS
>P0DUB9
ADTYAATRYPVILVHGLAGTDKFANVVDYWYGIQSDLQSHGAKVYVANLSGFQSDDGPNGRGEQLLAYVKQVLAATGATKVNLIGHSQGGLTSRYVAAVAPQLVASVTTIGTPHRGSEFADFVQDVLKTDPTGLSSTVIA
AFVNVFGTLVSSSHNTDQDALAALRTLTTAQTATYNRNFPSAGLGAPGSCQTGAATETVGGSQHLLYSWGGTAIQPTSTVLGVTGATDTSTGTLDVANVTDPSTLALLATGAVMINRASGQNDGLVSRCSSLFGQVISTS
YHWNHLDEINQLLGVRGANAEDPVAVIRTHVNRLKLQGV
SSSSSSSSSSSSSSSSSSSSSSSSSSSSSSSSSSSSSSSSSSSSSSSSSSSSSSSSSSSSSSSSSSSSSSSSSSSSSSSSSSSSSSSSSSSSSSSSSSSSSSSSSSSSSSSSSSSSSSSSSSSSSSSSSSSSSSSS
SSSSSSSSSSSSSSSSSSSSSSSSSSSSSSSSSSSSSSSSSSSSSSSSSSSSSSSSSSSSSSSSSSSSSSSSSSSSSSSSSSSSSSSSSSSSSSSSSSSSSSSSSSSSSSSSSSSSSSSSSSSSSSSSSSSSSSS
SSSSSSSSSSSSSSSSSSSSSSSSSSSSSSSSSSSSSSS
>P58486
MRSAQVYRWQIPMDAGVVLRDRRLKTRDGLYVCLRDGEREGWGEISPLPGFSQETWEEAQTALLTWVNDWLQGSEGLPEMPSVAFGASCALAELTGVLPEAADYRAAPLCTGDPDDLVLRLADMPGEKIAKVKVGLYEAV
RDGMVVNLLLEAIPDLHLRLDANRAWTPLKAQQFAKYVNPDYRARIAFLEEPCKTRDDSRAFARETGIAIAWDESLREADFTFEAEEGVRAVVIKPTLTGSLDKVREQVAAAHALGLTAVISSSIESSLGLTQLARIAAW
LTPGTLPGLDTLHLMQAQQIRPWPGSALPCLKREELERLL
SSSSSSSSSSSSSSSSSSSSSSSSSSSSSSSSSSSSSSSSSSSSSSSSSSSSSSSSSSSSSSSSSSSSSSSSSSSSSSSSSSSSSSSSSSSSSSSSSSSSSSSSSSSSSSSSSSSSSSSSSSSSSSSSSSSSSSSS
SSSSSSSSSSSSSSSSSSSSSSSSSSSSSSSSSSSSSSSSSSSSSSSSSSSSSSSSSSSSSSSSSSSSSSSSSSSSSSSSSSSSSSSSSSSSSSSSSSSSSSSSSSSSSSSSSSSSSSSSSSSSSSSSSSSSSSSS
SSSSSSSSSSSSSSSSSSSSSSSSSSSSSSSSSSSSSSSS
>Q1D4I1
MKKRVGIEALAVAVPSRYVDIEDLARARGVDPAKYTAGLGAREMAVTDPGEDTVALAATAAARLIRQQDVDPSRIGMLVVGTETGIDHSKPVASHVQGLLKLPRTMRTYDTQHACYGGTAGLMAAVEWIASGAGAGKVAV
VVCSDIARYGLNTAGEPTQGGGAVALLVSEQPDLLAMDVGLNGVCSMDVYDFWRPVGRREALVDGHYSITCYLEALSGAYRGWREKALAAGLVRWSDALPGEQLARIAYHVPFCKMARKAHTQLRLCDLEDAADAAASTP
ESREAQAKSAASYDAQVATSLGLNSRIGNVYTASLYLALAGLLQHEAGALAGQRIGLLSYGSGCAAEFYSGTVGEKAAERMAKADLEAVLARRERVSIEEYERLMKLPADAPEAVAPSPGAFRLTEIRDHRRQYAEGN
SSSSSSSSSSSSSSSSSSSSSSSSSSSSSSSSSSSSSSSSSSSSSSSSSSSSSSSSSSSSSSSSSSSSSSSSSSSSSSSSSSSSSSSSSSSSSSSSSSSSSSSSSSSSSSSSSSSSSSSSSSSSSSSSSSSSSSSS
SSSSSSSSSSSSSSSSSSSSSSSSSSSSSSSSSSSSSSSSSSSSSSSSSSSSSSSSSSSSSSSSSSSSSSSSSSSSSSSSSSSSSSSSSSSSSSSSSSSSSSSSSSSSSSSSSSSSSSSSSSSSSSSSSSSSSSSS
SSSSSSSSSSSSSSSSSSSSSSSSSSSSSSSSSSSSSSSSSSSSSSSSSSSSSSSSSSSSSSSSSSSSSSSSSSSSSSSSSSSSSSSSSSSSSSSSSSSSSSSSSSSSSSSSSSSSSSSSSSSSSSSSSSSSSS
>O43924
MSAKDERAREILRGFKLNWMNLRDAETGKILWQGTEDLSVPGVEHEARVPKKILKCKAVSRELNFSSTEQMEKFRLEQKVYFKGQCLEEWFFEFGFVIPNSTNTWQSLIEAAPESQMMPASVLTGNVIIETKFFDDDLLV
STSRVRLFYV

SSSSSSSSSSSSSSSSSSSSSSSSSSSSSSSSSSSSSSSSSSSSSSSSSSSSSSSSSSSSSSSSSSSSSSSSSSSSSSSSSSSSSSSSSSSSSSSSSSSSSSSSSSSSSSSSSSSSSSSSSSSSSSSSSSSSSSSSSSSS
SSSSSSSSSS
>Q5SL35
MRGIVTIDGPSASGKSSVARRVAAALGVPYLSSGLLYRAAAFLALRAGVDPGDEEGLLALLEGLGVRLLAQAEGNRVLADGEDLTSFLHTPEVDRVVSAVARLPGVRAWVNRRLKEVPPPFVAEGRDMGTAVFPEAAHKF
YLTASPEVRAWRRARERPQAYEEVLRDLLRRDERDKAQSAPAPDALVLDTGGMTLDEVVAWVLAHIRR
SSSSSSSSSSSSSSSSSSSSSSSSSSSSSSSSSSSSSSSSSSSSSSSSSSSSSSSSSSSSSSSSSSSSSSSSSSSSSSSSSSSSSSSSSSSSSSSSSSSSSSSSSSSSSSSSSSSSSSSSSSSSSSSSSSSSSSSSSSS
SSSSSSSSSSSSSSSSSSSSSSSSSSSSSSSSSSSSSSSSSSSSSSSSSSSSSSSSSSSSSSSSSSSS
>P0A6R0
MYTKIIGTGSYLPEQVRTNADLEKMVDTSDEWIVTRTGIRERHIAAPNETVSTMGFEAATRAIEMAGIEKDQIGLIVVATTSATHAFPSAACQIQSMLGIKGCPAFDVAAACAGFTYALSVADQYVKSGAVKYALVVGSD
VLARTCDPTDRGTIIIFGDGAGAAVLAASEEPGIISTHLHADGSYGELLTLPNADRVNPENSIHLTMAGNEVFKVAVTELAHIVDETLAANNLDRSQLDWLVPHQANLRIISATAKKLGMSMDNVVVTLDRHGNTSAASV
PCALDEAVRDGRIKPGQLVLLEAFGGGFTWGSALVRF
SSSSSSSSSSSSSSSSSSSSSSSSSSSSSSSSSSSSSSSSSSSSSSSSSSSSSSSSSSSSSSSSSSSSSSSSSSSSSSSSSSSSSSSSSSSSSSSSSSSSSSSSSSSSSSSSSSSSSSSSSSSSSSSSSSSSSSSSSSS
SSSSSSSSSSSSSSSSSSSSSSSSSSSSSSSSSSSSSSSSSSSSSSSSSSSSSSSSSSSSSSSSSSSSSSSSSSSSSSSSSSSSSSSSSSSSSSSSSSSSSSSSSSSSSSSSSSSSSSSSSSSSSSSSSSSSSSSSSS
SSSSSSSSSSSSSSSSSSSSSSSSSSSSSSSSSSSSS
>P48147
MLSLQYPDVYRDETAVQDYHGHKICDPYAWLEDPDSEQTKAFVEAQNKITVPFLEQCPIRGLYKERMTELYDYPKYSCHFKKGKRYFYFYNTGLQNQRVLYVQDSLEGEARVFLDPNILSDDGTVALRGYAFSEDGEYFA
YGLSASGSDWVTIKFMKVDGAKELPDVLERVKFSCMAWTHDGKGMFYNSYPQQDGKSDGTETSTNLHQKLYYHVLGTDQSEDILCAEFPDEPKWMGGAELSDDGRYVLLSIREGCDPVNRLWYCDLQQESSGIAGILKWV
KLIDNFEGEYDYVTNEGTVFTFKTNRQSPNYRVINIDFRDPEESKWKVLVPEHEKDVLEWIACVRSNFLVLCYLHDVKNILQLHDLTTGALLKTFPLDVGSIVGYSGQKKDTEIFYQFTSFLSPGIIYHCDLTKEELEPR
VFREVTVKGIDASDYQTVQIFYPSKDGTKIPMFIVHKKGIKLDGSHPAFLYGYGGFNISITPNYSVSRLIFVRHMGGILAVANIRGGGEYGETWHKGGILANKQNCFDDFQCAAEYLIKEGYTSPKRLTINGGSNGGLLV
AACANQRPDLFGCVIAQVGVMDMLKFHKYTIGHAWTTDYGCSDSKQHFEWLVKYSPLHNVKLPEADDIQYPSMLLLTADHDDRVVPLHSLKFIATLQYIVGRSRKQSNPLLIHVDTKAGHGAGKPTAKVIEEVSDMFAFI
ARCLNVDWIP
SSSSSSSSSSSSSSSSSSSSSSSSSSSSSSSSSSSSSSSSSSSSSSSSSSSSSSSSSSSSSSSSSSSSSSSSSSSSSSSSSSSSSSSSSSSSSSSSSSSSSSSSSSSSSSSSSSSSSSSSSSSSSSSSSSSSSSSSSS
SSSSSSSSSSSSSSSSSSSSSSSSSSSSSSSSSSSSSSSSSSSSSSSSSSSSSSSSSSSSSSSSSSSSSSSSSSSSSSSSSSSSSSSSSSSSSSSSSSSSSSSSSSSSSSSSSSSSSSSSSSSSSSSSSSSSSSSS
SSSSSSSSSSSSSSSSSSSSSSSSSSSSSSSSSSSSSSSSSSSSSSSSSSSSSSSSSSSSSSSSSSSSSSSSSSSSSSSSSSSSSSSSSSSSSSSSSSSSSSSSSSSSSSSSSSSSSSSSSSSSSSSSSSSSSSSSSS
SSSSSSSSSSSSSSSSSSSSSSSSSSSSSSSSSSSSSSSSSSSSSSSSSSSSSSSSSSSSSSSSSSSSSSSSSSSSSSSSSSSSSSSSSSSSSSSSSSSSSSSSSSSSSSSSSSSSSSSSSSSSSSSSSSSSSSSSSSSS
SSSSSSSSSSSSSSSSSSSSSSSSSSSSSSSSSSSSSSSSSSSSSSSSSSSSSSSSSSSSSSSSSSSSSSSSSSSSSSSSSSSSSSSSSSSSSSSSSSSSSSSSSSSSSSSSSSSSSSSSSSSSSSSSSSSSSSSSSSSS
SSSSSSSSSS
>Q2CEE3
MLTGVIEGFYGRDWRRDERATVMDWIAAAGMNTYIYGPKDDVHVRARWRVPYDAAGLARLTELRDAAAARGMVFYVSLAPCLDVTYSDPQDRAALLARVDQLARAGLRNLVLLFDDIPSVLPEADRHRFDSFAEAQADLS
NMVLRHLRGAGHVVFCPTEYCGRMAGGDPRGSAYLQRLGSTLDPAIDIFWTGPEIVSEEIVAAHLAAVGEVLRRRPVIWDNFHANDYDIRRVFAGPLGGRSRDILPLVAGWITNPNNEAEANFPAIHTTGAYLADPDYAP
ERAIAAAVAAWQPRFRLAFGDGAVPSDLVALLCDLFWQPFALGPETTRILSALRAALTVPRPDPSDPAWRAALEDLRDLKRRINKLFTLMTEIENRDLFHTFHNYLWEAQEEVGHLVAYCDWLDEAPPPGAVFPATDRIH
NFYRRGFGVAVQDILQRDRQGRYHHGV
SSSSSSSSSSSSSSSSSSSSSSSSSSSSSSSSSSSSSSSSSSSSSSSSSSSSSSSSSSSSSSSSSSSSSSSSSSSSSSSSSSSSSSSSSSSSSSSSSSSSSSSSSSSSSSSSSSSSSSSSSSSSSSSSSSSSSSSSSS
SSSSSSSSSSSSSSSSSSSSSSSSSSSSSSSSSSSSSSSSSSSSSSSSSSSSSSSSSSSSSSSSSSSSSSSSSSSSSSSSSSSSSSSSSSSSSSSSSSSSSSSSSSSSSSSSSSSSSSSSSSSSSSSSSSSSSSSS
SSSSSSSSSSSSSSSSSSSSSSSSSSSSSSSSSSSSSSSSSSSSSSSSSSSSSSSSSSSSSSSSSSSSSSSSSSSSSSSSSSSSSSSSSSSSSSSSSSSSSSSSSSSSSSSSSSSSSSSSSSSSSSSSSSSSSSSSSS
SSSSSSSSSSSSSSSSSSSSSSSSSSS
